# Assessing the Impact of Adlayer Description Fidelity on Theoretical Predictions of Coking on Ni(111) at Steam Reforming Conditions

**DOI:** 10.1021/acs.jpcc.3c02323

**Published:** 2023-04-27

**Authors:** Sai Sharath Yadavalli, Glenn Jones, Raz L. Benson, Michail Stamatakis

**Affiliations:** † Thomas Young Centre and Department of Chemical Engineering, University College London, Roberts Building, Torrington Place, London WC1E 7JE, U.K.; ‡ Johnson Matthey Technology Centre, Sonning Common, Reading RG4 9NH, U.K.

## Abstract

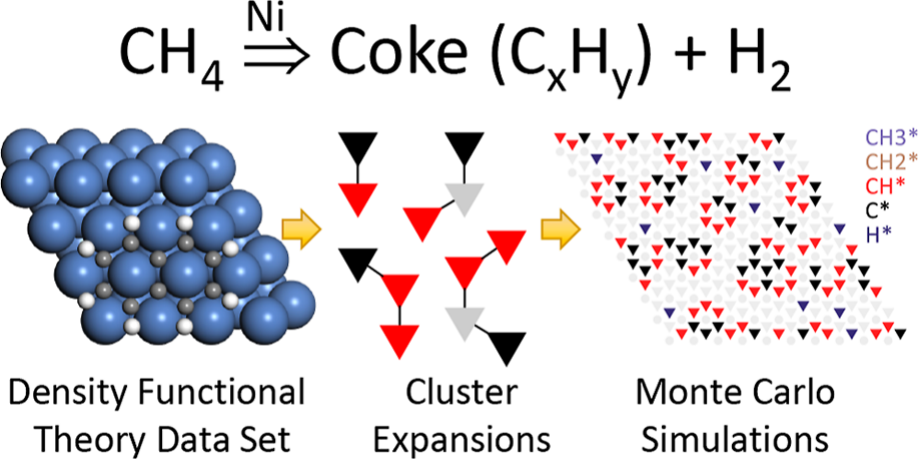

Methane steam reforming is an important industrial process for hydrogen production, employing Ni as a low-cost, highly active catalyst, which, however, suffers from coking due to methane cracking. Coking is the accumulation of a stable poison over time, occurring at high temperatures; thus, to a first approximation, it can be treated as a thermodynamic problem. In this work, we developed an Ab initio kinetic Monte Carlo (KMC) model for methane cracking on Ni(111) at steam reforming conditions. The model captures C–H activation kinetics in detail, while graphene sheet formation is described at the level of thermodynamics, to obtain insights into the “terminal (poisoned) state” of graphene/coke within reasonable computational times. We used cluster expansions (CEs) of progressively higher fidelity to systematically assess the influence of effective cluster interactions between adsorbed or covalently bonded C and CH species on the “terminal state” morphology. Moreover, we compared the predictions of KMC models incorporating these CEs into mean-field microkinetic models in a consistent manner. The models show that the “terminal state” changes significantly with the level of fidelity of the CEs. Furthermore, high-fidelity simulations predict C–CH island/rings that are largely disconnected at low temperatures but completely encapsulate the Ni(111) surface at high temperatures.

## 1 Introduction

The efficient production of hydrogen at the industrial scale is critical to meet the global energy demands of the 21st century.^1,2^ Hydrogen is widely used in various commercial processes such as methanol production, hydrogenation of unsaturated fats and oils, and manufacture of ammonia and sulfur removal from hydrocarbon streams.^3−6^ Furthermore, there is tremendous interest in the scientific community to use hydrogen as an alternative fuel (clean energy source) in vehicles and electricity generation to address global climate issues.^7−9^ In chemical industries, hydrogen is mainly produced via partial oxidation of methane, methane steam reforming (MSR), and dry reforming of methane.^10−13^ Among these industrial processes, MSR is cost-effective and contributes significantly to the production of hydrogen. In the United States, 95% of hydrogen production is achieved via the MSR process.^2,10^ Thus, it is crucial to sustain/improve the efficiency of the MSR process for ensuring high production rates of hydrogen at the industrial scale.

Although noble metals such as Pt, Pd, Ru, and Rh have been reported to exhibit good catalytic activity/stability for reforming processes,^14,15^ Ni is the preferred choice for MSR in the industry due to its low price, availability, acceptable activity, and selectivity.^2,10,16^ However, at steam reforming conditions, Ni-based catalysts are susceptible to deactivation—this severely hampers/hinders the productivity of the MSR process.^10,17,18^ Ni catalysts undergo deactivation mainly by three processes: (1) sulfur poisoning, (2) coking, and (3) sintering.^19−22^ Among these deactivation processes, coking has been found to have a deleterious impact on the performance of Ni. The coking process mainly involves deposition of carbonaceous species, which have been found to exist in the form of pyrolytic carbon, encapsulating carbon (gums), and carbon whiskers. The pyrolytic carbon is formed on the tubular walls of the reformer upon exposure of heavier hydrocarbons to high temperatures. Encapsulating carbon involves the deposition of CH_*x*_-type film on the Ni catalyst surface—this mainly occurs when the feed contains high amounts of aromatic carbon. Whisker carbon (also known as “filamentous carbon”) is the most destructive form of coke.^19,23,24^ The carbon formed in MSR binds to the step sites of the Ni catalyst initially and then migrates to the support side and agglomerates in the form of graphitic layers (carbon whiskers).^23,25^ Whisker carbon formation leads to significant reduction of Ni catalyst activity, increase in pressure drop, and reactor blockage.^23,26,27^ It is vital to prevent whisker carbon growth on Ni to improve the efficacy of the MSR process.

In the last few decades, several experimental studies have been carried out to gain a fundamental understanding on the growth mechanism of whisker carbon. It is generally accepted that the methane cracking [CH_4_(g) → C + 2H_2_(g)] and Boudouard [2CO → C + CO_2_(g)] reactions are primarily responsible for the growth of whisker carbon on the Ni catalyst surface at MSR conditions.^26,28,29^ Snoeck et al.^26^ performed extensive experiments to derive a kinetic model that accounts for the growth of carbon whiskers in the methane cracking reaction. Apart from the surface reactions of methane cracking, the authors included three additional elementary steps to describe the whisker carbon growth of methane cracking: (1) the dissolution/segregation of carbon from the Ni surface (gas side) into Ni bulk, (2) the diffusion of carbon through Ni bulk to the support side, and (3) the precipitation of carbon in the form of whiskers on the support side. It was hypothesized that at the coking threshold, the rate of whisker carbon formation is determined by the concentration difference between carbon gas and carbon bulk, while the diffusion of carbon from the bulk phase to the support side is fast. The authors also assumed that there is a uniform concentration of carbon at the Ni bulk and support side. Under these assumptions, a kinetic model was derived for the methane cracking reaction. The rate parameters of the model were determined by fitting to experimental data using parameter estimation techniques.^26^ Other studies have also used a similar approach to determine the coking propensity of Ni in the methane cracking reaction at steam reforming conditions.^11,30−32^ Although these studies provide useful insights into the whisker carbon growth of Ni, the coking process is far more complex. In order to gain deeper understanding of the coking phenomenon, it is imperative to develop fundamental models that capture thoroughly the thermodynamic stabilities of C_*x*_H_*y*_ species in the methane cracking reaction.^33^

In the past few decades, there has been considerable interest in the scientific community to use quantum chemistry approaches, such as density functional theory (DFT) calculations, to delineate the coking mechanism on the Ni catalyst surface. One of the seminal DFT works in this regard was conducted by Helveg and co-workers.^34^ The authors employed high-resolution in situ transmission electron microscopy (TEM) and DFT to elucidate the growth mechanism of whisker carbon due to methane decomposition on supported Ni nanocrystals. TEM imaging reveals that the carbon nucleation process involves the formation of graphene sheets on Ni(111) at the molecular level. Furthermore, the Ni surface undergoes dynamic restructuring to create steps/defects that enable the accumulation of carbon. Based on these observations, the authors hypothesized that graphene formation involves migration of carbon atoms from Ni steps/defects to the Ni(111) surface and subsequent carbon diffusion along the Ni(111) surface. A proposed DFT model of these processes was found to explain the TEM observations satisfactorily. Subsequently, Abild-Pedersen et al.^25^ performed detailed DFT studies to explore the thermodynamically favorable pathways for carbon migrations from steps/defects to the support side. These studies provide useful atomistic level insights into the coking mechanism on Ni. However, the intermediate steps/stages and plausible carbon poison precursors responsible for graphene formation on Ni(111) are not thoroughly explored, while it is critical to understand the growth of carbonaceous species on Ni(111) to mitigate/prevent graphene formation.^35^

Thus motivated, a few studies have presented DFT calculations aiming at elucidating the binding affinity of long-range carbon configurations such as chains, branches, and rings on Ni(111).^35−37^ Wang et al.^35^ calculated the binding energies of atomic carbon, carbon clusters (C_2_–C_4_), and graphene on Ni(111) and reported that graphene is thermodynamically the most stable configuration on Ni(111). Li et al.^37^ used DFT to find the optimized structures/energetics of carbon clusters such as chains, rings, and branches (containing up to six carbon atoms) on Ni(111) and concluded that carbon chains have better stability than rings/branches. The aforementioned DFT studies do not account for thermal, entropic, and coverage effects, which are important to thoroughly understand the coke formation due to the methane cracking reaction at MSR conditions.^33,38^ Recently, Li et al.^39^ developed a first principles-based KMC model for methane cracking on Ni(111), in the context of exploring the growth of carbon nanotubes (CNTs) on Ni. While the model shed light on the key role of surface species diffusion on CNT growth, effective cluster interactions (ECIs) between carbon-based intermediates were not taken into account.

Traditionally, DFT-parameterized microkinetic (MK) models are employed to study the reaction kinetics of catalytic systems.^40,41^ MK models predict important macroscopic observables of interest, such as turnover rates and species coverages at any given reaction condition. In the MK model formulation, mean-field approximations are typically used to account for adsorbate–adsorbate interactions.^42^ Several studies in the past have used mean-field MK models to capture the intrinsic kinetics and carbon poisoning chemistry under reforming conditions.^2,10,11,43^ Although mean-field approximations, within the MK framework, adequately take into account adsorbate interactions in some catalytic systems, they usually fail to capture short-/long-range correlations, clustering of adsorbates, lattice inhomogeneities, and island formation.^42,44,45^ In the methane cracking reaction, these effects might play a vital role in the accumulation of coke on Ni. Indeed, the need to systematically capture adsorbate correlation effects of carbon-based species has previously been highlighted in the context of gaining a detailed understanding of the catalyst poisoning at reforming conditions.^2,33^

Kinetic Monte Carlo (KMC) simulations have gained significant prominence, as a viable alternative to mean-field MK models, for studying catalytic reactions that involve high species coverages or occur under poisoning conditions. The cluster expansion (CE) methodology implemented in KMC provides a highly accurate description of adsorbate correlation effects, thereby allowing us to capture in detail the chemistry of complex catalytic reactions.^40,44^ Recently, several studies have successfully used CE-based KMC models to rationalize experimental findings of catalytic reactions.^44,46−48^ For instance, Piccinin and Stamatakis^47^ developed a CE parameterized KMC model for the CO oxidation reaction on oxygen pre-covered Pd(111). The authors were able to explain the apparent change in reaction order of CO oxidation at different reaction conditions (observed experimentally) by systematically accounting for oxygen–oxygen interactions under the CE methodology. Wu et al.^48^ developed CE-based metropolis Monte Carlo model for oxygen on Pt(111) to obtain qualitative agreement with experimental apparent activation energy and rate orders for the NO oxidation reaction. Thus, the CE-based KMC models can be a powerful tool to capture correlation effects of convoluted reactions.^49^

Coking is by definition the accumulation of a very stable poison over time, and for the MSR reaction, it happens in the context of a high-temperature process. Thus, to an acceptable first approximation, we are dealing with a thermodynamic problem, not a kinetic one. At long time scales, the most thermodynamically stable species will cover the Ni(111) surface, and thus, under the CE framework, we can capture the detailed energetics of such species to understand the formation of carbon-rich adlayers and identify the conditions where the emergence of coke is favorable. In this work, we developed an Ab initio KMC model for the methane cracking reaction on Ni(111), the primary reaction responsible for coke formation (as discussed previously). The model captures in detail the kinetics of the C–H activation steps, while graphene sheet formation is described at the level of thermodynamics, so as to obtain insights into the “terminal state” resulting in catalytic surface poisoning within reasonable computational times. We systematically explored the implications of including ECI effects in the methane cracking reaction to understand the terminal state of coke on Ni(111). In the KMC model, we have not considered the diffusion of carbon into the bulk and subsequent precipitation to form Ni carbide. As discussed earlier, previous experimental studies have argued that the growth of graphene/coke mainly occurs by carbon surface diffusion/agglomeration on the Ni support side,^34,50,51^ and DFT calculations have also shown that the diffusion barriers to Ni subsurface (around 1.34 eV) and Ni bulk (1.6–1.8 eV) are high.^39^ The rest of the article is organized as follows. In Section 2—Methods, we provide a thorough discussion about the methods employed in this study, and in Section 3—Results and Discussion, the results of the DFT calculations and kinetic simulations (using the MK and KMC approaches) are presented in a systematic way. Finally, in Section 4—Summary and Conclusions, we provide a detailed discussion on the conclusions/implications of this study and the potential opportunities for future work.

## 2 Methods

### 2.1 DFT Calculations

We performed spin-polarized plane wave DFT calculations using the Vienna Ab initio Simulation Package (VASP) 5.4.1. The tolerance value for the electronic (self-consistency) convergence was set to 10^–7^ eV. It is well known that there is a wide variation/uncertainty in the DFT predictions based on the choice of the exchange–correlation approximation.^33,43,52^ In our previous work, we performed detailed screening studies to identify a suitable DFT functional for the MSR–graphene system on Ni(111). We found the PBE-D3^53^ functional to be an appropriate choice for studying the carbon poisoning chemistry on Ni(111).^54^ Thus, the PBE-D3 approximation has been employed to capture the exchange–correlation effects in this study. The plane wave energy cut-off value was set to 400 eV (the plane wave convergence tests are available in the Supporting Information of our earlier work),^54^ and the interactions between core and valence electrons were modeled using the projector augmented wave potentials.

For the Ni lattice constant optimization calculations, the tetrahedron method with Blöchl corrections was employed to perform the electron smearing (the smearing width was set to 0.05 eV), and the Brillouin zone was sampled with a 19 × 19 × 1 *k*-point mesh. The optimized Ni lattice constant was thus found to be 3.481 Å (which is in reasonable agreement with the experimental Ni lattice constant—3.524 Å).^55^ The Ni(111) surface has been modeled using a six-layer *p*(4 × 4) slab (which has a vacuum height of 12 Å). The Ni atoms of the three bottom-most layers were fixed to their respective bulk positions, and the rest were fully relaxed until the Hellmann–Feynman forces reached a value of less than 10^–2^ eV/Å. In the Ni slab calculations, the electrons were smeared by employing the Methfessel–Paxton method (with a smearing width value of 0.1 eV), and the Brillouin zone sampling was performed using a 5 × 5 × 1 Monkhorst–Pack *k*-point grid (refer to the Supporting Information of our previous work for the *k*-points convergence plots).^54^

The geometric optimization of the adsorbates was conducted by employing the conjugate gradient search algorithm. The transition states (TSs) were located by using the dimer^56^ and quasi-Newton methods. The coordinates of the converged TS structures reported by Blaylock et al.^10^ were used as an initial guess (the coordinates of atoms were rescaled to account for the slightly different lattice constants between the two calculation setups). The vibrational frequencies of the converged structures were obtained by evaluating the Hessian matrix with the central finite difference method and a step size for the displacement of 0.02 Å. As shown in Table S2 of the Supporting Information, all the TS structures have a single imaginary mode, which indicates that these are first-order saddle points on the potential energy surface.

The formation energies of the adsorbates were computed using Ni(111) clean slab, CH_4_ (g), and H_2_ (g) as a reference (refer to eq 1 below).
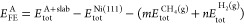
1
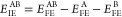
2In eq 1, *E*_tot_^Ni(111)^ refers to the DFT total energy of the Ni(111) slab, *E*_tot_^A+slab^ is the DFT total energy of the adsorbate-Ni(111) system, *E*_tot_^CH_4_(g)^ represents the gas-phase DFT total energy of methane, *E*_tot_^H_2_(g)^ indicates the gas-phase DFT total energy of hydrogen, and *E*_FE_^A^ is the formation energy of the adsorbate. The stoichiometry between the adsorbate and the gas-phase reference species (that is the number of C/H atoms) is balanced out using the real numbers *m* and *n*. For instance, the formation energy calculation of the CH adsorbate would have *m* and *n* values as 1 and −1.5, respectively. As shown in eq 2, the interaction energy for any co-adsorbed pair of species A and B (*E*_IE_^AB^) is obtained by subtracting the formation energies at infinite separation (the terms *E*_FE_^A^ and *E*_FE_^B^) from the co-adsorbed state formation energy (*E*_FE_^AB^).

### 2.2 Mean-Field Microkinetic Model

An elementary event involves the transition of the system from one particular potential energy surface (PES) basin to another. During this transition, the molecule spends a significant amount of time undergoing random vibrations. In kinetic models, the trajectory of the system is coarse-grained into discrete state-to-state “hops”. The time evolution of the system is thus governed by a Markovian master equation.^57^ The MK methodology can be derived by reducing the master equation into a system of ordinary differential equations (ODEs) under the assumption of infinitely fast adsorbate diffusions and large lattice size, whereby the correlation effects between adsorbates are neglected. Thus, the information about the spatial distribution of adsorbates on the lattice is lost within the MK framework.^58−60^

The methane cracking reaction involves 10 elementary events (as shown in Table 2). All reactions are assumed to be reversible. As shown in eqs 3 and 4, the reaction rate for each elementary event is given by the mass-action law expression (which involves the rate constants and species coverages). In eqs 3–5, *k* refers to the rate constant, *R*_*j*_^surf^ is the set of reactant species of the reaction, *P*_*j*_^surf^ is the set of product species of the reaction, θ represents the surface coverage (normalized with respect to the number of three-fold hollow sites), GM_fac_ is a “geometry factor” (accounting for site connectivity), *v*_*ij*_ is the stoichiometric coefficient of species *i* in the reactant/product set of reaction *j*, and *R*_*j*_^(*m*)^ represents the overall rate of reaction *j*. The system of ODEs is represented using eq 6. The rate of change in θ_*i*_ is given by the summation of rates of formation/consumption of species in each reaction multiplied by the corresponding stoichiometric coefficient. The Bragg–Williams (BW) approximation is employed to account for the interactions between adsorbates or covalently bonded species in the MK model.^41^ The interaction effect is represented as a product of the geometry factor, mean coverage, and interaction parameter (refer to eq 7). In eq 7, *E*_*i*_^FE^ indicates the formation energy, *E*_*i*_^FE-cov^ is the coverage-dependent formation energy, and *E*_*ij*_^1NN^, *E*_*ij*_^2NN^, and *E*_*ij*_^3NN^ are the first nearest neighbor, second nearest neighbor, and third nearest neighbor interaction parameters, respectively (refer to Section 2.5 for more details about how these interactions are included in the BEP relation). The MK model equations have been solved numerically using the ODE 15s solver in MATLAB 2019b.
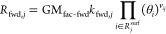
3
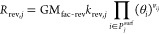
4
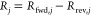
5
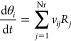
6
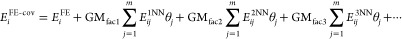
7

### 2.3 Kinetic Monte Carlo Simulations

The KMC approach does not deliver an explicit solution of the Markovian master equation; rather, it employs a stochastic simulation algorithm to generate trajectories whose statistics follow this equation. The observables of interest can be obtained by time-averaging these stochastic realizations (trajectories) upon reaching steady-state conditions.^58,61^ In this work, the KMC simulations have been carried out by using graph-theoretical KMC software Zacros 3.01.^62^

The preferred binding sites of methane cracking adsorbates are recorded in Table S1 of the Supporting Information. We have chosen a KMC lattice which comprises top and three-fold hollow sites (where fcc and hcp sites are considered identical). In Figure S2 of the Supporting Information, the KMC lattice is depicted. The circles and triangles (which are colored in blue for vacant sites) represent the top and hollow sites, respectively. A lattice of size 10 × 10 has been used to run the KMC simulations (lattice convergence results are shown in Table S8). In order to reduce the computational cost of KMC simulations, the pre-exponentials of quasi-equilibrated (fast) events are downscaled systematically by carefully analyzing the event occurrence statistics plot throughout the run time of the KMC simulation (the frequency of events is checked for a sliding interval of 5 × 10^–1^ KMC time units).

The interactions between adsorbates or covalently bonded species have been captured by employing the CE methodology implemented in the graph-theoretical KMC framework by Nielsen et al.^63^ According to the CE formalism, the formation energy of a configuration is expanded as a sum of interaction energies of clusters/figures (refer to eq 8).^48,63^ The clusters (also called as “patterns”) to be included in the CE are identified/selected by following a hierarchical approach by which all *k*-1 body clusters contained in a *k*-body cluster must be included in the CE before incorporating that *k*-body cluster.^64^ In eq 8, *H*(σ) is the formation energy of a lattice configuration, ECI_*k*_ refers to the effective cluster interaction of pattern *k*, NOC_*k*_ is the number of times the pattern *k* is identified in a configuration, and GM_*k*_ is the graph-multiplicity of pattern *k* (this multiplicity factor is included to avoid double counting of symmetric clusters in the graph-theoretical KMC framework).^63^
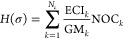
8

In principle, we can include all the possible 1-body, 2-body ···. *n*-body clusters/figures in the CE model to have an exact representation of energy in the KMC simulation. However, this procedure becomes increasingly tedious and computationally expensive for a large data set of DFT configurations.^65^ This problem can be addressed by truncating the CE model using a finite set of optimal clusters/figures, which are obtained by performing CE-based least-squares fitting. The identification and parameterization of clusters in the CE fitting exercise are a non-trivial task.^66^ It involves trial and error, and the decision on when the CE is accurate enough involves the use of metrics such as root-mean-square-error (RMSE) and leave-one-out-cross-validation (LOOCV) score (this provides a statistical measure of the CE model predictive accuracy).^66,67^
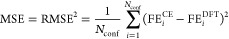
9
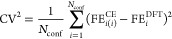
10In eq 9, the formula for obtaining MSE/RMSE is shown. FE_*i*_^CE^ denotes the formation energy of the *i*th configuration, as predicted by the CE (using the entire data set of *N*_conf_ configurations), and FE_*i*_^DFT^ is the formation energy calculated from DFT. Equation 10 gives the formula for computing the CV score, with FE_*i*(*i*)_^CE^ denoting the formation energy of configuration *i*, when configuration *i* is omitted from the data set used for the CE fitting. For more details, we refer the reader to the work by Miller and Kitchin,^64^ which includes a discussion and an application of the LOOCV methodology in the context of CE fitting. Briefly, a low LOOCV score ensures high-quality fit of CE parameters.^67^ In some cases, the CE model’s predictive capability is also benchmarked against experimental data (if available). Furthermore, it is crucial to identify the appropriate number of clusters as the use of too many clusters in the CE model can lead to overfitting issues.^64^

**Figure 1:**
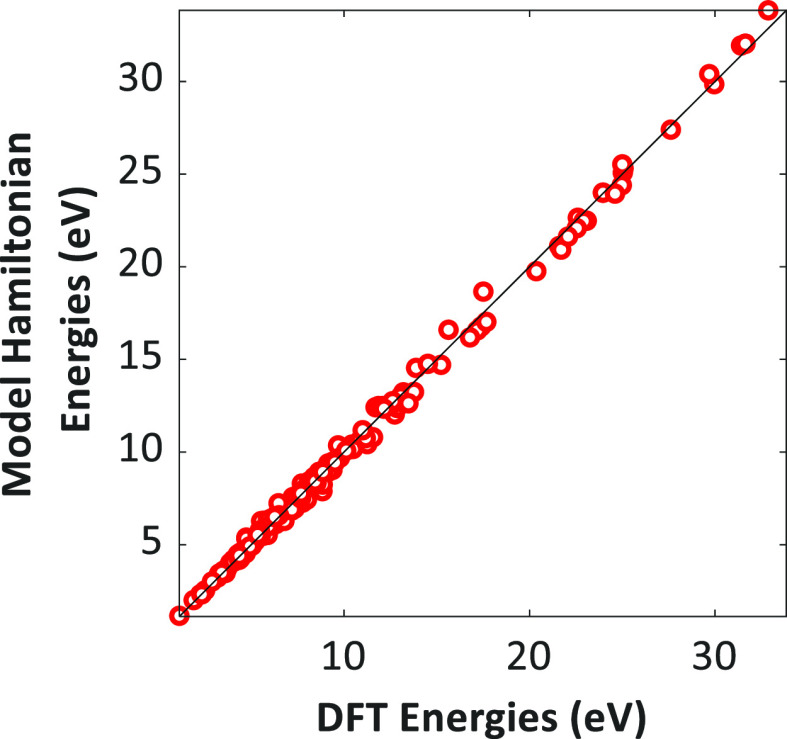
Parity between the CE model and DFT energies of C/CH configuration data set.

The pairwise ECI parameters, as estimated using eq 2, are recorded in Table S6 of the Supporting Information. In Figure S2, a schematic for each type of the pairwise interaction pattern is provided. We also performed a CE optimization for a data set of 173 unique DFT configurations on 4 × 4 supercells. The data set mainly comprises rings, branches, and chain-type configurations of CH/C species (more details are provided in the “Results and Discussion” section). The statistical metrics of the CE fit are provided in Table 1—these are within the acceptable limits.^64,67^ The parity between CE and DFT predictions is depicted in Figure 1. Table S7 of the Supporting Information shows the ECI values of the CE-fit parameters/figures. In Figures S3–S5 of the Supporting Information, the schematics of the parameters/figures used for fitting the CE model are illustrated. Furthermore, the Cook’s distances^68^ were estimated for each configuration of the CE data set (refer to Figure S6 of the Supporting Information), in order to detect configurations that potentially exert a strong influence on the ECI values obtained from the regression. The Cook’s distance for the *i*th configuration has been calculated using the following equation
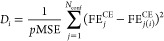
11where *p* is the number of parameters/figures of the CE, MSE represents the mean-squared error, and FE_*j*(*i*)_^CE^ is the formation energy of configuration *j* obtained after omitting configuration *i* in the data set of CE fitting.

**Table 1:** CE Optimization Metrics for the CH/C DFT Configurations

metrics	value
number of DFT configurations	173
number of parameters/figures	23
RMSE (meV/site)	52.65
LOOCV score (meV/site)	8.87

The 173 configurations used to fit the CE were constructed in a systematic manner. We started off with one-body, two-body, and three-body configurations of C and CH species and fitted the CE with simple parameters/figures. Then, we gradually expanded the data set to higher-body configurations and used a trial-and-error approach to identify the suitable parameters/figures for our CE model. We consistently used the metrics such as RMSE, CV score, and Cook’s distances to check the quality of the fit. If any of the metrics were unsatisfactory, we refitted the CE model by removing/including figures. Furthermore, whenever a highly influential configuration was found (as quantified by its Cook’s distance), we enriched the data set with configurations that contained similar motifs, thereby better sampling that region of the configuration space. The DFT data set includes configurations which have coverage ranging from 0 to 1 ML.

### 2.4 Establishment of Equivalence between MK and KMC Models

In order to make a systematic comparison between MK and KMC models, it is imperative to first obtain equivalent results. The equivalence condition for MK and KMC models is as follows: at the limit of fast diffusions and large system size, without interactions, both MK and KMC models give identical results.^69^ There are some “technicalities” involved in establishing equivalence between MK and KMC models; in particular, appropriate geometry factors (GM_fac_) must be included in the MK model equations (as shown in eqs 3 and 4) to account for site connectivity of the lattice. For events in which the reactant patterns are symmetric, the pattern detection algorithm of Zacros double counts the number of instances thereof on the lattice. Thus, the kinetic constants of such events must be corrected by dividing them with the “event-multiplicity” factor. The geometry and event-multiplicity factors for each reaction step of the methane cracking reaction network are provided in Table S5. As stated above, it is critical to ensure that the diffusion events are quasi-equilibrated (fast) to achieve equivalence between MK and KMC models. However, in certain scenarios (especially under high species coverage regimes), it might be necessary to include adsorbate swap diffusions in the KMC simulation to establish equivalence with MK predictions. These are concerted diffusion events which may not necessarily be physically realistic; their role is to ensure better homogenization of the KMC lattice under “crowded” (high species coverage) conditions.^69^ In our study, we have been able to obtain quantitatively similar results for the methane cracking reaction using MK and KMC models at the equivalence condition (more details are provided in the “Results and Discussion” section).

### 2.5 Estimation of Pre-exponentials and Activation Energy Parametrization

The first reaction step in the methane cracking reaction involves dissociative adsorption of methane to form methyl and hydrogen on the Ni(111) surface (as shown in Table S7). For an activated dissociative adsorption event, we calculate the forward/reverse rate constants using eqs 12 and 13 (as defined below). In these equations, *m*_CH_4__ is the mass of the methane molecule, *P*_CH_4__ represents the pressure of methane gas, *A*_st_ is the contact surface area of the Ni atom, *T* is the temperature, *k*_B_ is the Boltzmann constant, *E*_Actfwd_^finite-coverage^ represents the coverage-dependent forward activation energy, *E*_Actrev_^finite-coverage^ is coverage-dependent reverse activation energy, *Q*_trans-2D_ is the translational partition function of a 2D gas, and *Q*_rot_ and *Q*_vib_ are the rotational and vibrational partition functions, respectively.
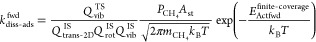
12
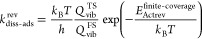
13
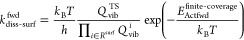
14
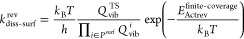
15In the case of surface reactions, the frustrated translations and rotations of chemisorbed species are considered as vibrations. The vibrational partition function is estimated by using the harmonic approximation. The forward/reverse rate constants of surface reactions are estimated using eqs 14 and 15 (as shown above). The forward/reverse coverage-dependent activation barriers for any reaction are calculated using the BEP relations (as shown in eqs 16 and 17 below) in MK and KMC models. In eqs 16 and 17, Δ*E*_rxn_ is the coverage-dependent reaction energy, Δ*E*_rxn_^0^ is the zero-coverage reaction energy, *w* is the proximity factor, and *E*_Act_^zero-coverage^ is the zero-coverage activation barrier. The proximity factor of each elementary event is listed in Table S5.
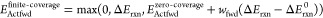
16
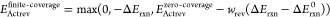
17

## 3 Results and Discussion

In this study, we attempted to elucidate the formation of carbon-based poisoning species on the Ni support surface by systematically accounting for adsorbate–adsorbate correlation effects in the methane cracking reaction (as discussed previously in the “Introduction” section). The methane cracking reaction is a highly correlated system. Thus, the inclusion of interactions between adsorbates or covalently bonded species in the methane cracking reaction can potentially provide us with useful information about the structure of coke/graphene on the Ni support surface. Moreover, it could lay the groundwork for more complicated models that capture in detail the growth kinetics of the various C_*x*_H_*y*_ coke precursors.

With these points in mind, the “Results and Discussion” section is structured as follows: we first discuss in detail the nature/magnitude of interactions between carbonaceous species (at 1NN, 2NN, and 3NN distances) and present the DFT data set of C/CH long-range configurations in Section 3.1. Next, we compare systematically the MK and KMC predictions of methane cracking and draw conclusions about the influence of interactions on the thermodynamic stability and macroscopic coverages of methane cracking species in Section 3.2. We subsequently demonstrate lattice configurations obtained from KMC simulations with varying levels of detail in the description of adlayer energetics and develop an understanding of the dependence of the terminal state (the structure of the adlayer at the poisoned state) on ECIs in Section 3.3. Finally, we illustrate, in Section 3.4, the effect of temperature on the KMC adlayer and process statistics.

### 3.1 DFT Calculations

The adsorbate binding energies (Table S1) and activation barriers (Table S3) of the methane cracking reaction are reported in the Supporting Information. As mentioned previously (in the “Methods” section), Table S6 provides the interaction energy values at 1NN, 2NN, and 3NN distances for all the possible pairs of methane cracking adsorbates. It can be inferred from Table S6 that there is substantial variation in the type of interaction, attractive or repulsive, between 1NN and 2NN pairs of adsorbates or covalently bonded species encountered in methane cracking. At the 1NN distance, most co-adsorbed configurations of methane cracking species are unstable (due to the presence of strong repulsive interactions)—for such adsorbate pairs, a penalty is introduced in the MK/KMC model by fixing the value of ECI to 5 eV (as shown in Table S6), to prevent such configurations from appearing during the course of the simulation. In the case of C and CH species, we observe that the interaction is strongly attractive at the 1NN level.

**Figure 2:**
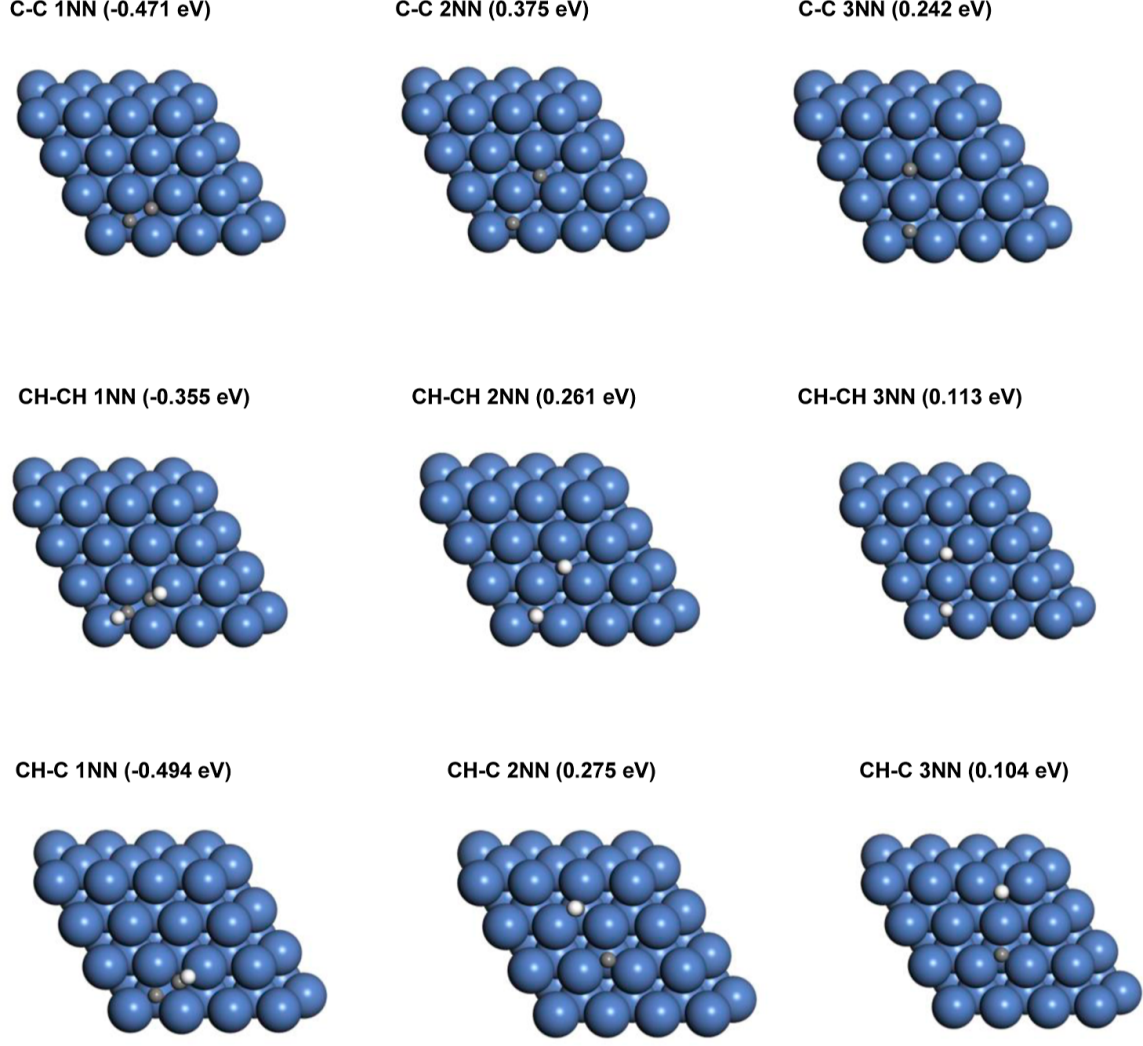
Top-view of the DFT configurations for the C/CH pairwise co-adsorbed states at 1NN, 2NN, and 3NN distances. The numbers in the parentheses are the ECI values of the corresponding patterns.

Thus, as shown in Figure 2, the ECI values of the C–C, C–CH, and CH–CH 1NN pairs are −0.471, −0.494, and −0.355 eV, respectively, which indicates that these pairs are highly stable on the Ni(111) surface. A few studies have also made similar observations about the C–C interactions.^36,37^ Li et al.^37^ performed projected density of states analysis of C–C species on Ni(111) and reported that there is an overlap of 2s and 2p orbitals of both carbon atoms, which is indicative of a strong C–C bond. The C/CH adsorbate–1NN pairs could be potential precursors to coke formation at steam reforming conditions.

In contrast to the attractive 1NN interactions of C/CH species, all the methane cracking species experience substantial repulsive interactions at the 2NN level (refer to Table S6). The C–C, CH–CH, and C–CH 2NN-adsorbate pairs have positive ECI values (refer to Figure 2). As shown in Table S6, most methane cracking 3NN–adsorbate pairs have a weaker repulsive interaction (the ECI values are converging to zero in some cases). At the 3NN distance, the interactions become less pronounced as the adsorbates are placed further apart from each other. It can be inferred from Figure 2 that the formation of long-range carbonaceous species on Ni(111) primarily involves an interplay of attractive (C–C, CH–CH, and C–CH bond formation at the 1NN level) and repulsive interactions (C–C, CH–CH, and C–CH repulsions at the 2NN/3NN level).

**Figure 3:**
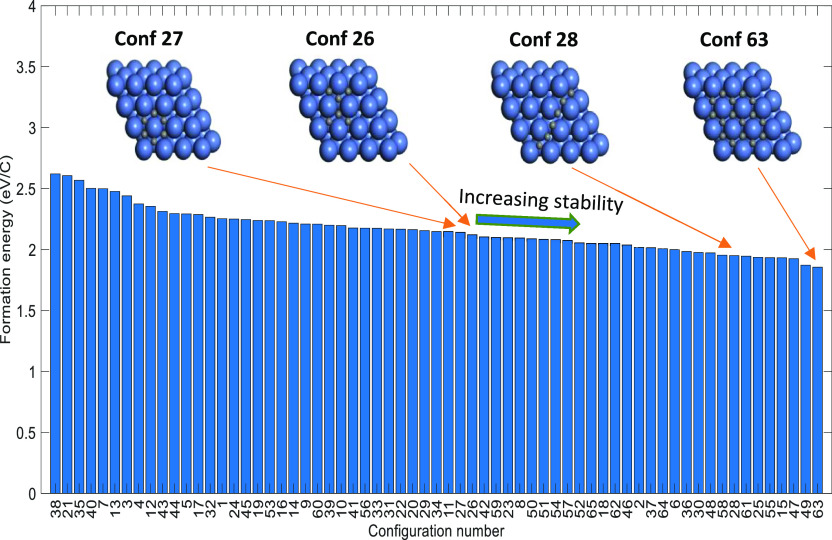
Formation energies (eV/C) of carbon configurations (reported in the increasing order of stability—from left to right), which are part of the DFT data set used for CE training.

**Figure 4:**
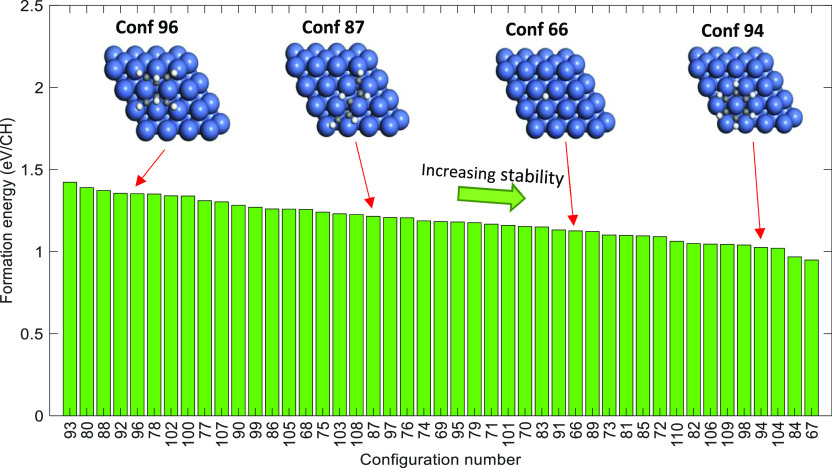
Formation energies (eV/CH) of CH configurations (reported in the increasing order of stability—from left to right), which are part of the DFT data set used for CE training.

**Figure 5:**
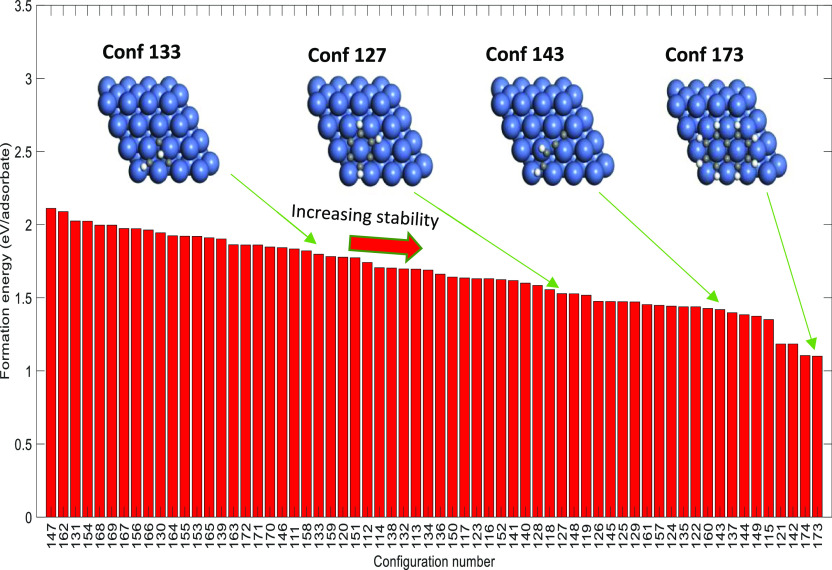
Formation energies (eV/adsorbate) of CH–C configurations, which are part of the DFT data set used for CE training.

We further explored the stability of long-range chains, branches, and rings (composed of C/CH species) on the Ni(111) surface. We performed DFT calculations to compute the formation energies of 173 different carbon, CH and CH–C configurations on Ni(111) as mentioned earlier in the “Methods” section. The data set has been developed in a systematic way; it includes a range of configurations at varying C/CH coverages (0–1 ML). The formation energies of carbon, CH, C–CH configurations are depicted in Figures 3–5, respectively.

It can be observed from Figure 3 that the formation energies of carbon configurations are in the range of 1.86–2.62 eV/C. As shown in Figure 3, among the six body configurations, the carbon chain has a formation energy of 1.95 eV/C (configuration 28), whereas the carbon branch and carbon ring configuration have a formation energy of 2.12 eV/C (configuration 26) and 2.14 eV/C (configuration 27), respectively. Li et al.^37^ and Cheng et al.^36^ also reported that the six-body chain-type carbon configurations have better stability than six-body rings/branches. Nevertheless, as depicted in Figure 3, the C16 ring-based (configuration 63) has the highest stability among the carbon configurations computed in this study, which indicates that the higher-body ring-based carbon structures could plausibly act as precursors to graphene/coke formation on Ni(111).

The formation energies of CH configurations lie in the range of 0.95–1.42 eV/CH (refer to Figure 4). The six-body CH ring, which is benzene (configuration 94), has far greater stability than most of the other CH configurations—this is mainly due to the π–π conjugation between carbon atoms in the benzene ring [the formation energy of benzene on Ni(111) is 1.03 eV/CH]. As shown in Figure 4, the five-body chain (configuration 87) has higher stability than the six-body branch-type CH configuration (configuration 96).

Furthermore, the C–CH configurations (Figure 5) have formation energies in the range of 1.10–2.11 eV/adsorbate (this is within the formation energy ranges of carbon and CH configurations). As illustrated in Figure 5, naphthalene (configuration 173) is very stable on the Ni(111) surface. Configurations 127 (partially hydrogenated carbon-based ring) and 143 (partially hydrogenated carbon-based chain) have formation energies 1.52 eV/adsorbate and 1.42 eV/adsorbate, respectively (these lie in the moderate range in terms of stability in the DFT data set). On the other hand, the C–CH branch-based structure (configuration 133) is less stable in comparison to other configurations (the formation energy value is 1.80 eV/adsorbate). Some of the CE training schematics of C–CH configurations are shown in Figures S7 and S8 of the Supporting Information. The complete data set of DFT configurations is available in the NOMAD repository.^70^ These calculations clearly indicate that C/CH correlation effects play a critical role in the formation of long-range carbonaceous species (which ultimately poison the Ni surface). Thus, it is important to systematically account for interactions between adsorbates or covalently bonded species to elucidate the poisoning chemistry of carbon-based species at steam reforming conditions.

### 3.2 Methane Cracking Reaction—MK/KMC Predictions

In an attempt to clearly understand the implications of interactions, we followed a systematic approach in developing the MK and KMC models for the methane cracking reaction. In Table 2, the list of elementary reactions of methane cracking along with their corresponding activation barriers, reaction energies, and pre-exponentials is provided. In the first instance, we attempted to obtain equivalence between MK and KMC models in the absence of interactions, and thus, appropriate geometry and event-multiplicity factors were included, as discussed previously in Section 2.4 of the methodology. Upon achieving this equivalence, we systematically started incorporating ECI parameters into both models.

**Table 2:** List of Elementary Events, Activation Barriers/Reaction Energies, and Pre-exponents of the Methane Cracking KMC Model at 1000 K and 10.01 bar^a^

event ID: reaction	*E*_Actfwd(rev)_^zero-coverage^ (eV)	Δ*E*_rxn_^0^ (eV)	pre-exp fwd (rev) (s^−1^)
R1: CH_4_(g) + *(fcc) + *(top) + *(fcc) → CH_3_*(fcc) + *(top) + H*(fcc)	0.41 (0.94)	−0.53	7.47 × 10^8^ (3.70 × 10^14^)
R2: CH_3_*(fcc) + *(top) + *(fcc) → CH_2_*(fcc) + *(top) + H*(fcc)	0.66 (0.64)	0.02	1.09 × 10^14^ (4.63 × 10^13^)
R3: CH_2_*(fcc) + *(fcc) → CH*(fcc) + H*(fcc)	0.26 (0.63)	−0.36	3.21 × 10^13^ (4.16 × 10^13^)
R4: CH*(fcc) + *(top) + *(fcc) → C*(fcc) + *(top) + H*(fcc)	1.31 (0.84)	0.46	1.92 × 10^14^ (1.14 × 10^14^)
R5: H*(fcc) + *(top) + H*(fcc) → H_2_(g) + *(fcc) + *(top) + *(fcc)	1.33 (0.00)	1.33	9.80 × 10^6^ (6.25 × 10^15^)

a Note: the activation barriers and reaction energies reported in this table do not include ZPE corrections. The reverse activation barriers/pre-exponentials of the corresponding reactions are shown in parentheses. The TS configurations of reactions R1, R2, R4, and R5 involve top sites of Ni(111), and thus, the KMC event definitions for these reactions include top sites. Please refer to Figure S1 (in the Supporting Information) for the TS geometries of these reactions.

The BW approximation (eq 7) was used to account for pairwise interactions up to 3NN level in the MK model. Three MK models were thus developed, namely, MK-1NN, which includes 1NN interactions, MK-1NN–2NN, which incorporates 1NN and 2NN interactions, and MK-1NN–2NN–3NN, which includes 1NN, 2NN, and 3NN interactions. The CE approach (eq 8) was used to account for interactions between adsorbates or covalently bonded species in the KMC model. We developed four different KMC models with increasing levels of complexity: the first three models, KMC-1NN, KMC-1NN–2NN, and KMC-1NN–2NN–3NN, include the interactions noted, as per the naming convention of the MK models just discussed (refer to Table S6 of the Supporting Information for ECI values). The fourth model, KMC-long-range, includes the pairwise interactions up to 3NN level, as well as the higher-level interactions, which are parameterized by fitting against the DFT data set that includes long-range carbon-based species (refer to Table S7 of the Supporting Information for ECI values).

**Figure 6:**
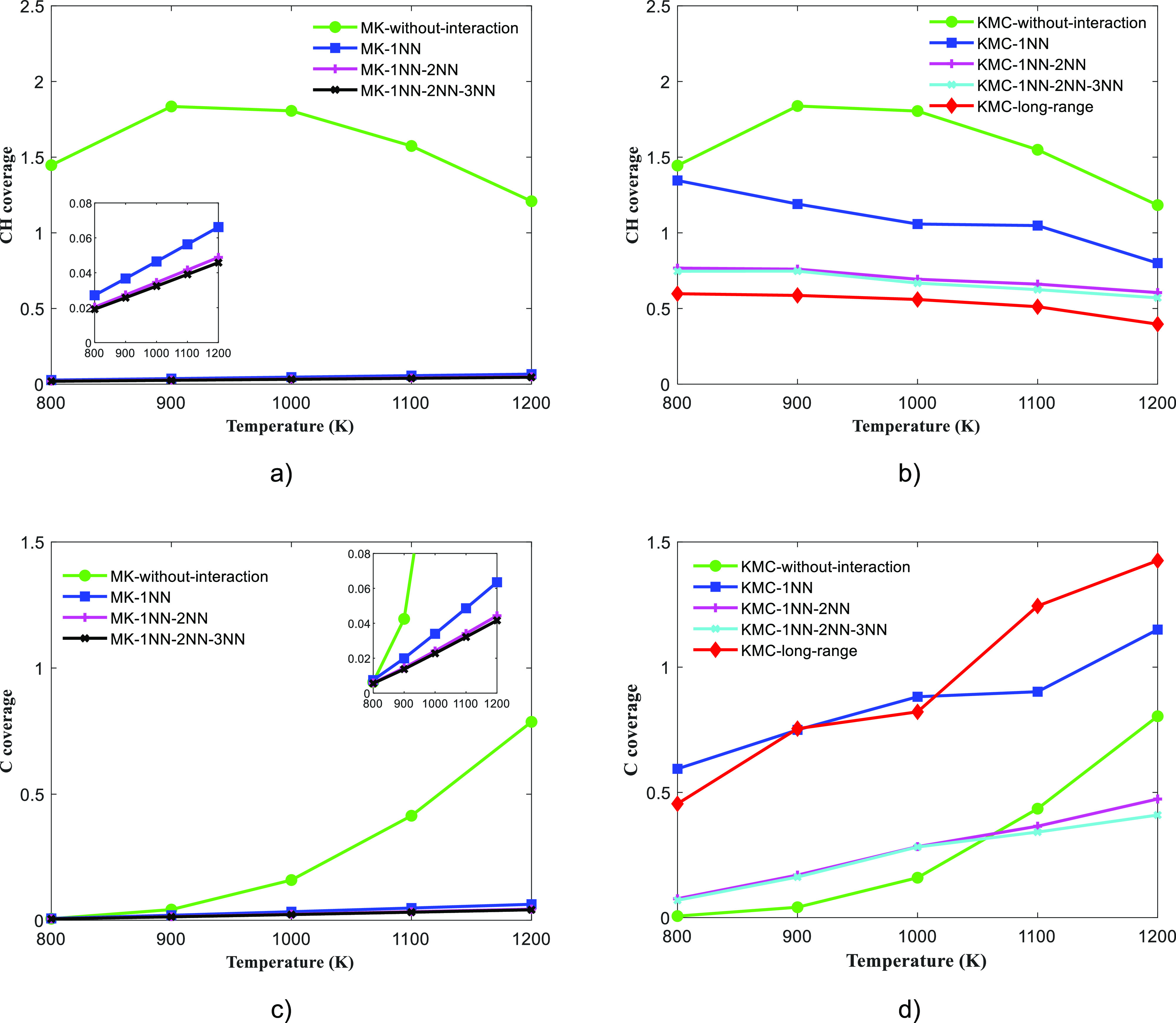
Coverage (ML) profiles with respect to temperature: (a) MK results of CH coverages, (b) KMC results of CH coverages, (c) MK results of carbon coverages, and (d) KMC results of carbon coverages. The methane and hydrogen pressure were maintained at 10.00 and 0.01 bar, respectively. The coverage is normalized with respect to number of surface Ni atoms, and thus, the maximum coverage is 2, when all hollow sites are covered.

The MK/KMC predictions were obtained at temperature ranges of 800–1200 K. The methane partial pressure and H_2_ partial pressure were maintained at 10.00 bar and 0.01 bar, respectively (in the gas phase). These are the typical industrial operating conditions of steam reforming,^10,12,15^ and Snoeck et al.^26^ also conducted experiments on the methane cracking reaction at similar conditions to investigate the carbon whisker growth. Figure 6a,b shows the CH coverage predictions of the MK and KMC models, respectively (at varying temperatures), while the carbon coverage predictions are depicted in Figure 6c,d, respectively.

In the absence of interactions, the MK and KMC models predict quantitatively identical coverage trends (refer to the “MK-without-interaction” and “KMC-without-interaction” model predictions in Figure 6). Without the inclusion of interactions in the MK/KMC models, the CH species is found to have high coverages on Ni(111) at steam reforming conditions (refer to Figure 6a,b). The surface dissociation steps following methane activation, i.e., the cleavage of CH_3_ and CH_2_ species, have high propensities, and thus, the CH_3_ and CH_2_ intermediates are short-lived on the Ni(111) surface. On the other hand, the CH dissociation step has a high free-energy barrier, and CH is thermodynamically the most stable species on the Ni(111) surface (refer to Tables S9–S13 of Supporting Information for the free-energy/kinetic data), which justifies the high coverage prediction of CH species at steam reforming conditions. As shown in Figure 6c,d, the MK-without-interaction and KMC-without-interaction models show an increasing trend of carbon coverage with respect to temperature. Although the CH dissociation event is endothermic, at higher temperatures, the formation of carbon is favored as kinetic effects become more pronounced (refer to Tables S9–S13 of Supporting Information for the free-energy/kinetic data). The inclusion of interactions between adsorbates or covalently bonded species in the MK and KMC model can substantially alter the thermodynamic stabilities of carbon intermediates of the methane cracking reaction (under the BEP relation)—this will be discussed in further detail below.

It is evident from Figure 6a,c that the MK models fail to capture the effect of interactions in a systematic fashion. The MK-1NN, MK-1NN–2NN, and MK-1NN–2NN–3NN models predict very low coverages of CH and C despite the inclusion of attractive interactions (CH–CH–1NN, CH–C–1NN, and C–C–1NN). The MK models tend to show small variation in CH and C coverages with respect to temperature. There is a significant difference between the predictions of MK and KMC models at all operating conditions (as shown in Figure 6, the difference lies in the range of 0.4–1 ML). Under the BW approximation, the likelihood of the occurrence of an adsorbate pair is determined by the geometry factor (accounting for site connectivity), the corresponding ECI value, and the averaged coverage. Since the spatial information of adsorbates is represented using averaged coverage in the BW methodology, the MK models provide an inaccurate estimate of the “average” number of CH/C interactions at any time step of the simulation. Thus, the mean-field MK models may not be reliable in understanding the growth mechanism of carbonaceous species at steam reforming conditions.

The KMC-1NN model predicts high CH and carbon coverages at all reaction conditions (800–1200 K). As discussed previously, the CH–CH, CH–C, and C–C interactions are attractive at the 1NN-level due to bond formation between C/CH species (refer to Table S6 for the ECI values). At steam reforming conditions, these attractive interactions increase the stability of CH/C species on Ni(111). We observe that the KMC-1NN model predicts far higher carbon coverages on Ni(111) in comparison to the “KMC-without-interaction” model (refer to Figure 6d). The coverage-dependent reaction energy term (Δ*E*_rxn_) of the CH dissociation event is lowered due to the inclusion of these attractive C–C and CH–C 1NN interactions, which in turn reduces the coverage-dependent forward activation barrier of the CH dissociation event as per the BEP relation (refer to eq 16). The improved thermodynamic stability of carbon species and reduction in coverage-dependent forward CH dissociation barrier favor the formation of carbon on the Ni(111) surface.

Upon inclusion of both 1NN and 2NN interactions, the KMC simulation predicts substantial CH (0.60–0.77 ML) and carbon coverages (0.07–0.47 ML) on Ni(111) at steam reforming conditions. However, the KMC-1NN–2NN CH/C coverage predictions are significantly lower than those of the KMC-1NN model (as shown in Figure 6b,d). As discussed previously, at the 2NN level, the carbon-based species experience substantial repulsive interactions (refer to Table S6 for ECI values at the 2NN level). These repulsive interactions decrease the overall thermodynamic stability of the CH/C adsorbates, which results in lower CH/C coverages on Ni(111).

Furthermore, as depicted in Figure 6b,d, the KMC-1NN–2NN–3NN model predicts C/CH coverages similar to those of the KMC-1NN–2NN model. At the 3NN level, the repulsions between adsorbates are weaker than that at 2NN distance as the adsorbates are further apart (refer to Table S6 for 3NN ECI values). Thus, the coverages of C/CH species are not significantly different from those of the KMC-1NN–2NN model.

Interestingly, upon capturing the detailed energetics of large body configurations (chains, branches, and rings) in the KMC simulation, we observe that carbon species tend to dominate over CH at higher temperatures (900 K and above). For instance, at 1200 K, the KMC-long-range model predicts the CH and carbon coverages to be 0.40 and 1.43 ML, respectively (refer to Figure 6b,d). This is at variance to the free energy/kinetic data in the absence of interactions (refer to Tables S9–S13 of the Supporting Information), according to which the CH species are more thermodynamically stable than carbon at steam reforming conditions. However, in the presence of long-range CH–CH, CH–C, and C–C interactions, the stability of carbon species improves dramatically, as demonstrated by the predictions of the KMC-long-range model.

Overall, the KMC models (with varying degree of accuracy in capturing adlayer energetics) have shown that the ECIs play a critical role in determining the overall thermodynamic stability and macroscopic coverages of the methane cracking species. These results give rise to several important questions: (1) does the surface morphology of Ni(111) change due to interactions? (2) what is the type/shape of carbon-based cluster that is thermodynamically stable on the Ni(111) surface? and (3) at what operating conditions is Ni more susceptible to coking/poisoning? In the subsequent sections, we will address these questions in detail.

### 3.3 Changes to the KMC Adlayer with Varying Levels of Interactions

**Figure 7:**
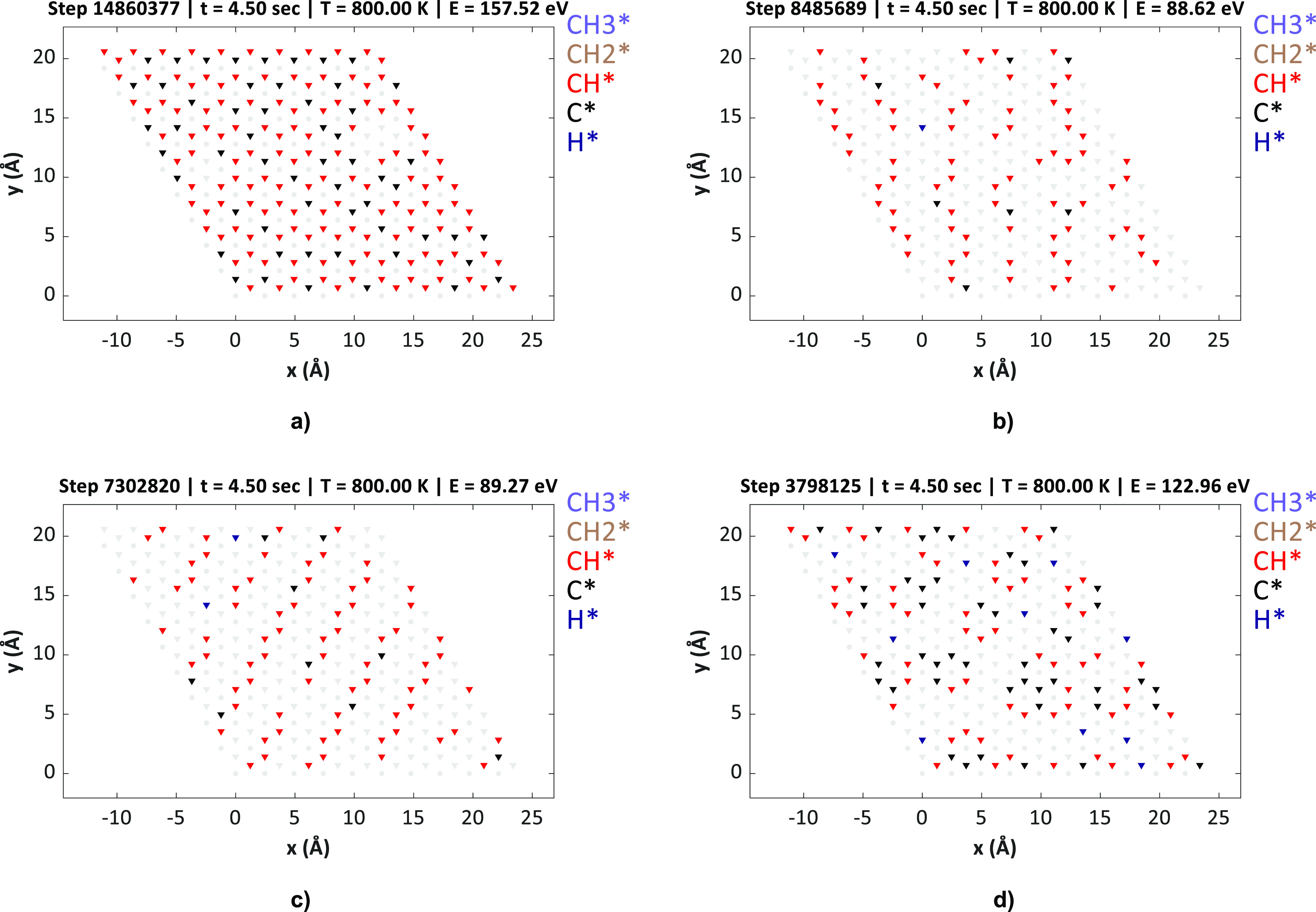
KMC lattice snapshots upon reaching steady state (poisoned condition) at 800 K. The CH_4_ and H_2_ pressures were maintained at 10.00 and 0.01 bar, respectively. (a) KMC-1NN, (b) KMC-1NN–2NN, (c) KMC-1NN–2NN–3NN, and (d) KMC-long-range.

In the previous discussion, we have observed that the reaction thermodynamics and macroscopic coverages of carbon-based species are significantly altered upon gradually refining the ECIs in the KMC simulations of methane cracking. Interactions between adsorbates or covalently bonded species, as captured via the CE methodology, give a better representation of the local environment at poisoning conditions, thereby allowing us to examine in detail the predictions of different CE-based models regarding the terminal state of coke on Ni(111). Thus motivated, Figure 7 provides the final lattice snapshots, for which the net rate of CH_4_ consumption/coking is close to zero (poisoned state), for the four KMC models. We assume that the system has reached the poisoned/terminal state when the species coverage fluctuations are within 0.02 ML.

The final lattice state of the KMC-1NN model is completely covered with CH/C species. Although there is no visible ordering of CH/C species into specific configurations, we do observe small clusters of carbon surrounded by three CH species throughout the KMC-1NN lattice (for instance, at *x* = 9 and *y* = 10 in Figure 7a). As shown in Figure 7b, upon including 2NN interactions in the system, we can observe mainly chain-based ordering of C/CH species in the final KMC lattice state. Similarly, the final lattice state of the KMC-1NN–2NN–3NN model also has CH and C species arranged in the form of straight chains (refer to Figure 7c).

The carbon atoms in the chain-based configurations appear to arrange themselves in such a way so as to minimize the number of 2NN and 3NN C–C, C–CH, and CH–CH interaction patterns, which are repulsive (refer to Table S6). Because of these repulsions, C/CH chains show higher stability than other configurations such as rings/branches, and the latter are hardly observed on the KMC lattice. Nevertheless, the aforementioned KMC models cannot accurately capture the formation energies of long-range chains, rings, and branches since the corresponding CEs do not include long-range (longer than 3NN) or many-body contributions.

Such contributions are included in the CE of the KMC-long-range model, whose final lattice state is significantly different from those of the other KMC models. As illustrated in Figure 7d, we find that the CH and carbon species form ring-based configurations. This is consistent with the DFT predictions of long-range CH and carbon configurations (as discussed in Section 3.1), which show that C–CH rings have higher thermodynamic stability on Ni(111) than chains/branches. Thus, surface coke could be composed of partially hydrogenated C–CH rings, which agglomerate to form long graphene sheets upon complete poisoning of the Ni support surface. Furthermore, a few experimental studies have shown that coke has a heterogeneous composition, plausibly containing large hydrocarbons, and the morphology and thermodynamic properties of coke differ considerably from a graphitic/nickel carbide phase.^71^ It is clear from the above discussion that the predicted morphology of the coke “terminal state” changes substantially based on the level of ECIs included in the KMC model.

### 3.4 Effect of Temperature on the KMC Adlayer and Process Statistics

**Figure 8:**
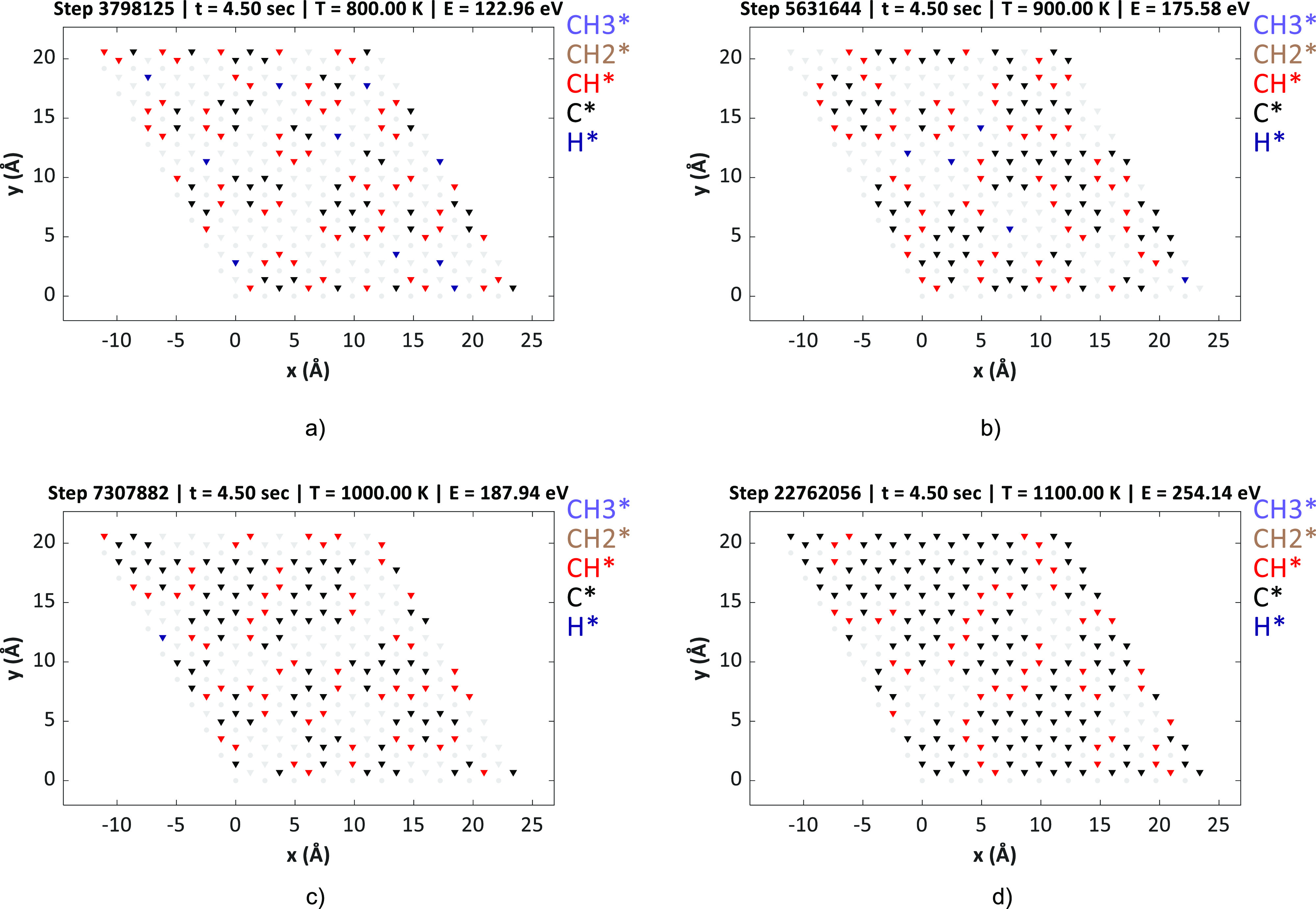
Lattice snapshots of the KMC-long-range model upon reaching steady state (poisoned condition) at varying temperatures. The CH_4_ and H_2_ pressures were maintained at 10.00 and 0.01 bar, respectively. (a) 800, (b) 900, (c) 1000, and (d) 1100 K.

The final lattice snapshots (poisoned state) of the KMC-long-range model are depicted at varying temperatures (800–1100 K) in Figure 8, which shows that at lower temperatures, the formation of CH/carbon rings is localized. Hence, we find mainly six-body ring configurations at specific regions, for instance, at 800 K, the rings are located at around the coordinates (2.5,15), (12.5,12.5), (22.5,15) Å, etc. These rings are C_6_H_*y*_-type configurations (where *y* varies from 1–4 in most cases) that are largely disconnected from each other. At moderate temperatures such as 900 and 1000 K, we observe the formation C13 and C16 ring-based super-clusters at various regions of the KMC lattice. On the other hand, at higher temperatures (1100 K and beyond), large islands of carbon-based rings completely cover the Ni(111) surface. The terminal points of these carbon islands are mostly populated with CH species. Based on these KMC simulations, we can conclude that graphene/coke is thermodynamically stable on Ni(111) at the operating conditions of MSR. The removal of these graphene/coke flakes from the Ni surface is difficult at the higher temperature regions of the steam reformer.

**Figure 9:**
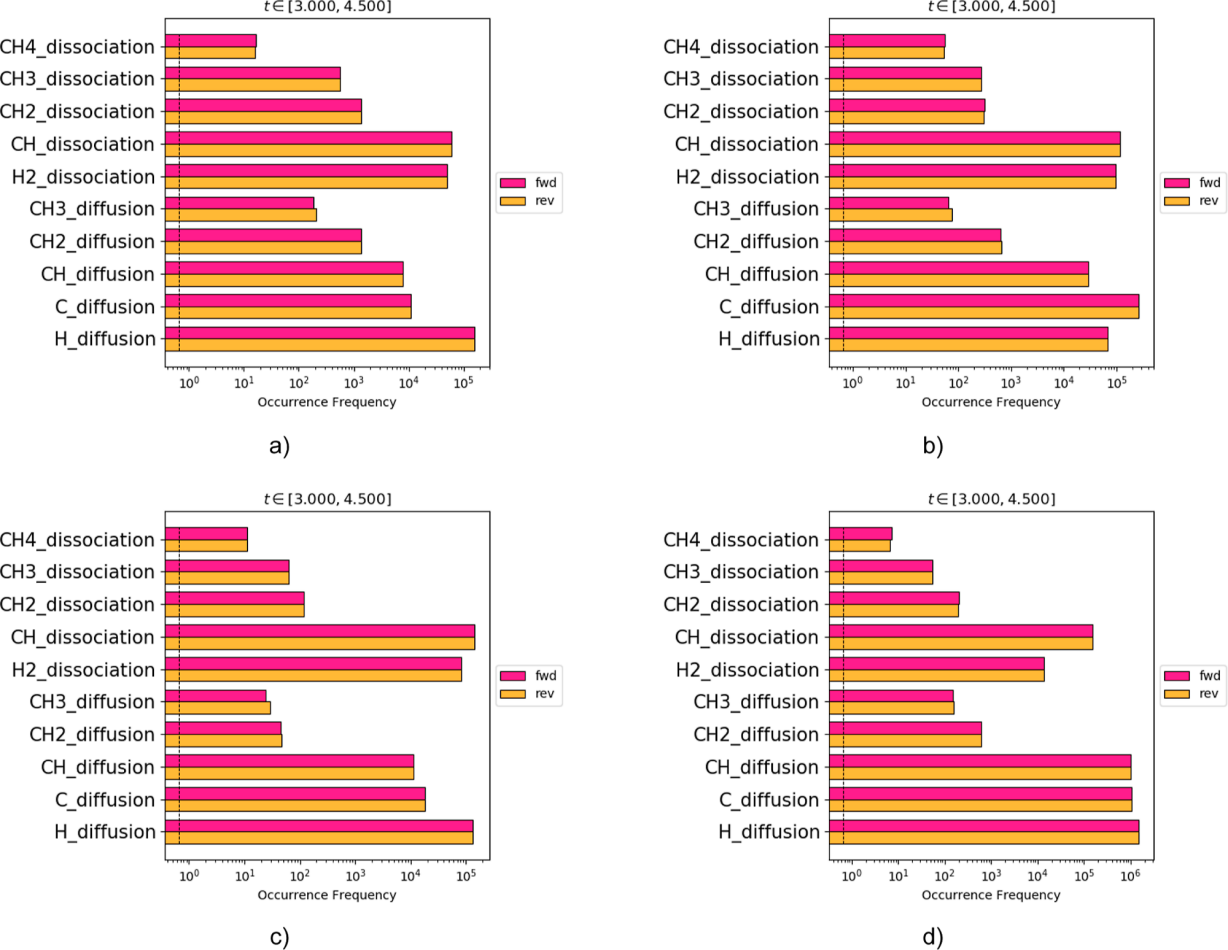
Reaction occurrence statistics of the KMC-long-range model at different temperatures: (a) 800, (b) 900, (c) 1000, and (d) 1100 K.

In Figure 9, the reaction occurrence statistics plot of the KMC-long-range model is depicted at various temperatures (800–1100 K). It is evident from these plots that out of the dehydrogenation steps included in our methane cracking model, methane dissociation (toward methyl and hydrogen) is the slow step at all temperatures since it has the lowest rate. This is in excellent agreement with other mean-field MK models present in the literature.^26^ Furthermore, the CH_2_ dissociation, CH dissociation, and H_2_ dissociation events are fast (quasi-equilibrated) at each operating condition. It is important to note, however, that our model captures only the thermodynamics of the formation of carbon-rich adlayer structures; thus, C–C coupling events are not explicitly considered and could potentially be rate-limiting at (some of) the conditions investigated. Developing a detailed model that takes into account these events is the subject of future research efforts.

## 4 Conclusions

The idea of using hydrogen as a clean energy source to meet global energy demands has gained immense popularity among the scientific community in the past few decades. MSR is an important contributor to the production of hydrogen at the industrial scale. However, the formation of coke (in the form of carbon whiskers) on the Ni catalyst surface severely hampers the productivity of MSR. The whisker carbon growth process mainly involves accumulation of carbonaceous species in the form of graphitic layers on the Ni support. It is well known that the methane cracking and Boudouard reactions are primarily responsible for whisker carbon formation at MSR conditions. Although several experimental studies have been carried out to elucidate whisker carbon growth due to methane cracking, there is little fundamental insights into the coking mechanism on Ni.

In recent years, first-principles methods such as DFT have been used to understand the growth of carbon whiskers at the atomistic and molecular levels. The DFT models developed thus far do not account for thermal and entropic effects, which are critical to understand coke formation at MSR conditions. Conventionally, mean-field MK models are employed to predict the kinetics of catalytic reactions. However, the mean-field approximations of MK models cannot capture adsorbate correlations and lattice inhomogeneities accurately; yet it is important to systematically account for these effects in reactions such as methane cracking to properly understand the growth mechanism of carbon whiskers. The CE-based KMC simulations capture interactions between adsorbates or covalently bonded species with high fidelity.

In this work, DFT calculations have revealed that there is significant variation in the nature and magnitude of interaction between 1NN and 2NN C/CH pairs. At the 1NN level, the C–C, C–CH, and CH–CH interactions are attractive due to overlap of p-orbitals leading to bond formation, whereas the 2NN adsorbate pairs of C/CH experience repulsive interactions. This indicates that the formation of long-range carbonaceous species on Ni(111) involves an interplay of C/CH attractions and repulsions. The many-body configurations of carbon-based species can take the form of chains, rings, and branches on Ni(111). Among the long-range CH configurations, the chains and rings have better stability than branched structures. This is consistent with other studies available in the literature. The correlations of C/CH species can play a crucial role in the initial growth of coke on Ni(111).

To thoroughly assess the consequences of interactions on the coke morphology, we developed MK and KMC models of the methane cracking reaction. In these simulations, the kinetics of subsequent dehydrogenations from CH_4_ to C + 4H are modeled in detail, while the formation of coke is captured at the level of thermodynamics only. Thus, C–C coupling events are not explicitly considered, but the stability of large carbon-rich islands and surface-layers/graphene is captured via the CE approach to a progressively higher level of accuracy. To this end, our KMC simulations incorporate the ECIs in an incremental fashion. The “zero interaction” MK and KMC models give quantitatively similar results. In the absence of interactions, the Ni(111) surface is predominantly covered with CH species. Upon inclusion of 1NN, 2NN, and 3NN interactions in the KMC model, we see a substantial change in the C/CH coverages and methane cracking reaction thermodynamics. MK models predict very low coverages of CH/C species upon inclusions of interaction terms. Since the spatial distribution of adsorbates is lost within the MK framework, the latter inaccurately predicts the average number of attractive/repulsive interactions at any time step of the simulation.

We further parameterized high-fidelity CEs using our DFT data set, thereby enabling the calculation of the formation energies of long-range carbon-based configurations (chains, rings, and branches) on Ni(111) during KMC simulations. The resulting KMC-long-range model includes the 1NN, 2NN, and 3NN interactions as well as the many-body ECIs. In contrast to the lower fidelity KMC models (KMC-1NN, KMC-1NN–2NN, and KMC-1NN–2NN–3NN), the KMC-long-range model predicts carbon to be the dominant species on Ni(111) at MSR conditions. The final lattice snapshot of the KMC-long-range model of the methane cracking reaction clearly shows that CH/C species accumulate on Ni(111) in the form of rings. These observations are consistent with the calculated DFT energetics of long-range configurations. The growth of carbonaceous species seems to involve formation of C–CH ring-based structures, which might branch together at higher coverages to form graphene sheets/coke.

The DFT data set used for CE training only comprised C, CH, and CH–C configurations that occupied the three-fold hollow sites. In future efforts, the data set can be further enriched by including carbon-based configurations that occupy top sites as well, and the KMC model can be enhanced by taking into account C–C coupling steps explicitly. These would be important to gain a thorough understanding of graphene growth (as the most stable configuration of graphene is top-fcc).^54,72^ A multi-faceted KMC model (that includes step sites) can also be developed to capture the migration mechanism of carbon from Ni step edge to Ni terrace—this could provide a more complete picture of the Ni catalyst deactivation. The multifaceted KMC model can be compared to relevant experimental studies of methane cracking available in the literature.^73^ Furthermore, the burn-off/oxidation mechanism of the carbon-based poison from the Ni catalyst surface could be of great industrial interest (in the context of Ni catalyst regeneration). Overall, the CE parameterized KMC simulations have delivered a better understanding on the coke/graphene “terminal state” at steam reforming conditions as they capture correlation effects with high fidelity. Our study paves the road toward future simulations which could potentially help us identify the next-generation Ni-based catalysts that are more resistant to coking.

## Supporting Information Available

Site preferences and binding energies of methane cracking adsorbates; vibrational wavenumbers of converged geometries; DFT configurations of the initial and TSs of the methane cracking system; schematics of the KMC lattice with the interaction patterns used in the CEs; comparison of computed activation barriers for the elementary steps of the methane cracking reaction against literature values; comparison of methane dissociation activation barriers using different DFT functionals against available experimental data; geometry, event-multiplicity, and proximity factors for each elementary event of methane cracking; Cook’s distances for the DFT-parameterized CE model; ECI values for the patterns used in the CEs; schematics of representative configurations of the DFT data set containing C and CH used for CE training; results of the KMC lattice convergence test; and free energy and kinetic data of methane cracking for a temperature range between 800 and 1200 K (PDF)
