# Supplementary material for: Assessing the Impact of Adlayer Description Fidelity on Theoretical Predictions of Coking on Ni(111) at Steam Reforming Conditions
Source: J Phys Chem C Nanomater Interfaces. 2023 Apr 27;127(18):8591–606. doi: 10.1021/acs.jpcc.3c02323 (PMC10184169; doi:10.1021/acs.jpcc.3c02323)
Supplement: Supplementary file 1 — jp3c02323_si_001.pdf [file jp3c02323_si_001.pdf]

Supplementary Information

Assessing the Impact of Adlayer Description Fidelity on Theoretical Predictions of Coking on Ni(111) at Steam Reforming Conditions

Sai Sharath Yadavalli[1], Glenn Jones[2], Raz L. Benson[1] and Michail Stamatakis[1*]

[1] Thomas Young Centre and Department of Chemical Engineering, University College London, Roberts Building, Torrington Place, London WC1E 7JE, United Kingdom

[2] Johnson Matthey Technology Centre, Sonning Common, Reading RG4 9NH, United Kingdom

[*] e-mail: m.stamatakis@ucl.ac.uk

Table S1. The site preferences and the binding energies of methane cracking adsorbates

Species	Preferred binding sites	Binding energy (eV)	Literature values (eV)
CH_4*	Top	-0.26	-0.02[1]
CH_3*	Fcc	-2.32	-1.91[1]
CH_2*	Fcc	-4.33	-4.01[1]
CH*	Fcc	-6.71	-6.43[1]
C*	Hcp	-7.01	-6.78[1]
H*	Fcc	-2.93	-2.81[1]
H_2*	Top	-0.49	-0.22[1]

Note: simulation details of Ref. [1]: p(3x3) Ni(111) supercell, ENCUT: 400 eV, k-point mesh: 5×5×1, PBE functional.

Table S2. The vibrational wavenumbers of converged geometries

System	Vibrational wavenumbers (cm^{-1})
CH_3*	2831, 2831, 2765, 1285, 1285, 1165, 480, 479, 400, 360, 225, 224
CH_2*	3010, 2351, 1431, 664, 591, 532, 360, 311, 282
CH*	3031, 656, 656, 629, 418, 418
C*	580, 540, 540
H*	1150, 859, 858
H_2*	2433, 1698, 925, 293, 246, 81
CH_4 dissociation TS	3105, 3067, 2962, 1567, 1391, 1372, 1139, 837, 725, 386, 363, 135, 91, 34, 899i
CH_3 dissociation TS	3008, 2514, 1851, 1369, 863, 707, 537, 451, 324, 267, 131, 850i
CH_2 dissociation TS	3054, 1207, 881, 677, 610, 467, 415, 360, 585i
CH dissociation TS	1858, 561, 522, 518, 128, 811i
H_2 dissociation TS	1905, 1691, 395, 213, 193, 532i

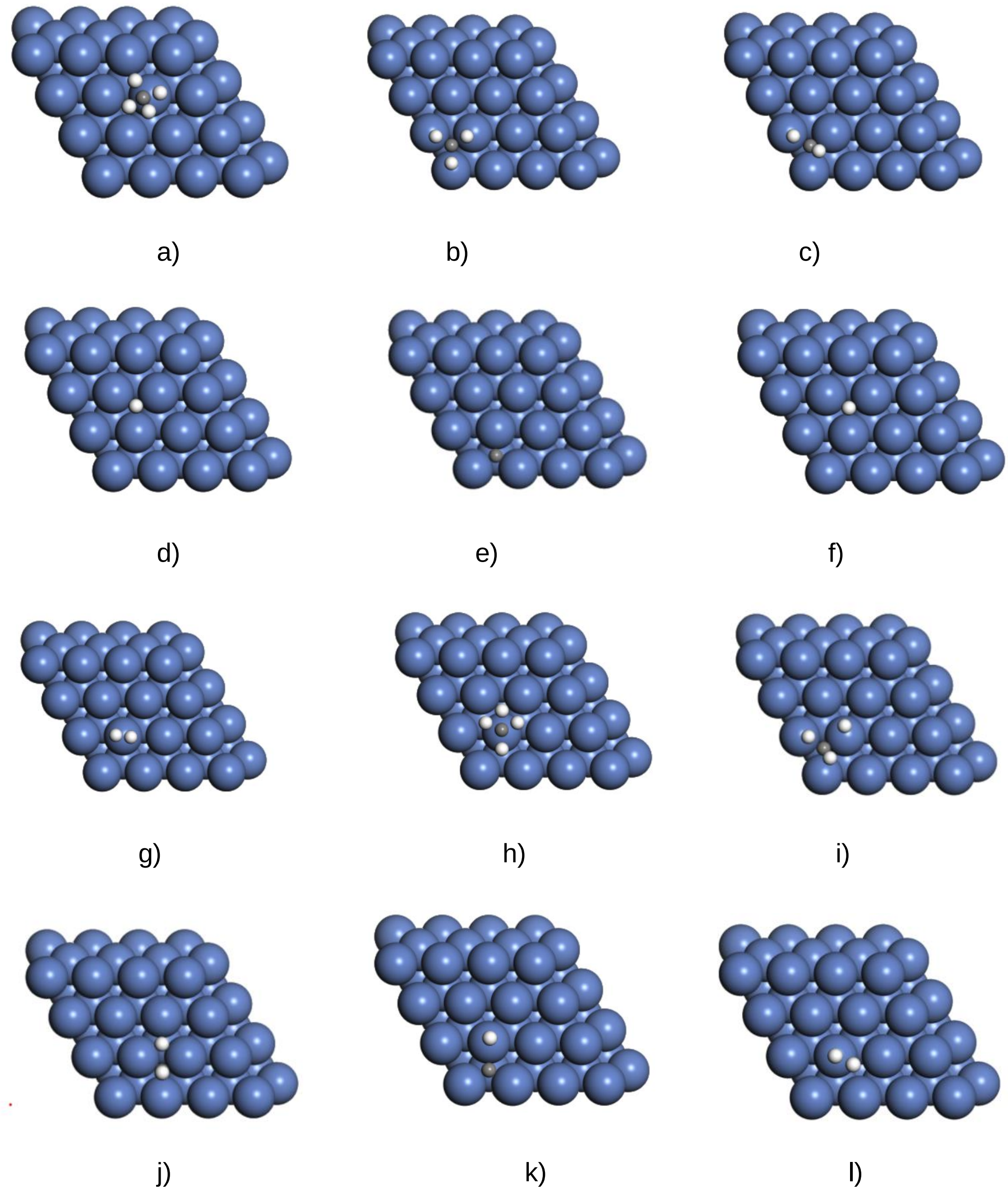

Figure S1: Top view of the initial states and transition states of the methane cracking system on Ni(111): a) CH_4 physisorbed state on the top site. b) CH_3 binds on the fcc site. c) CH_2 adsorbs on the fcc site. d) CH binds on the fcc site. e) Carbon has a preference to bind on the hcp site. f) Hydrogen prefers to bind on the fcc site. g) H_2 physisorbs on the top site. h) Transition state (TS) of methane dissociation. i) TS of CH_3 dissociation. j) TS of CH_2 dissociation. k) TS of CH dissociation. l) TS of H_2 dissociation.

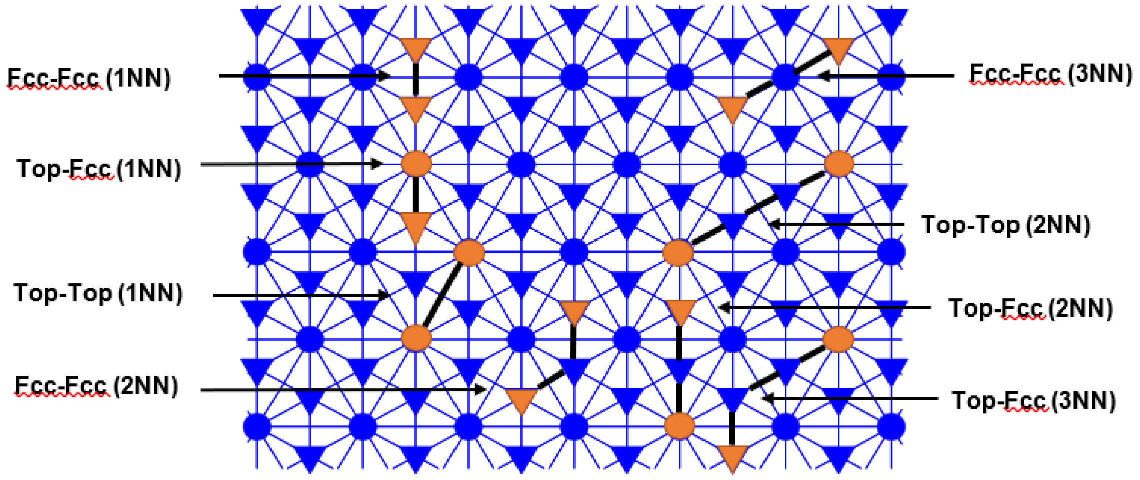

Figure S2: Schematic of the KMC lattice with all the pairwise interaction sub-patterns. The circles and triangles represent top and hollow sites, respectively. The unoccupied sites are coloured in blue, whereas the occupied sites are coloured in orange (the occupied site could be any adsorbate of the methane cracking system). Each type of interaction pattern has been given an appropriate label (as depicted above).

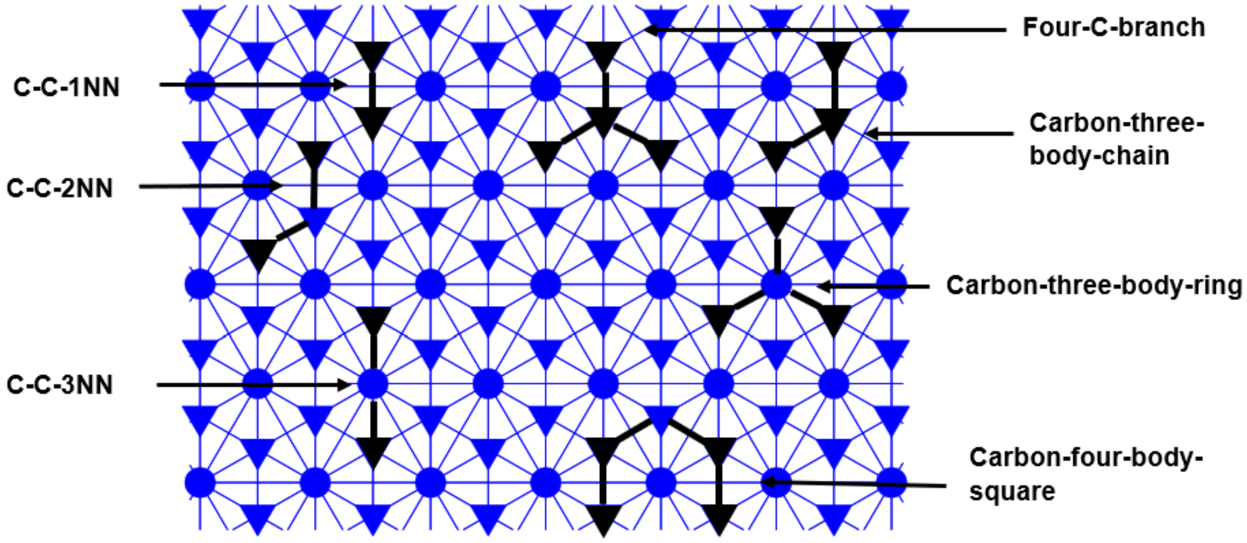

Figure S3: Schematics of interaction patterns used in the cluster expansion (CE) for capturing long-range carbon configurations. The circles and triangles represent top and hollow sites, respectively. The unoccupied sites are coloured in blue, whereas the occupied sites by carbon are coloured in black. Each type of interaction pattern has been given an appropriate label (as shown above).

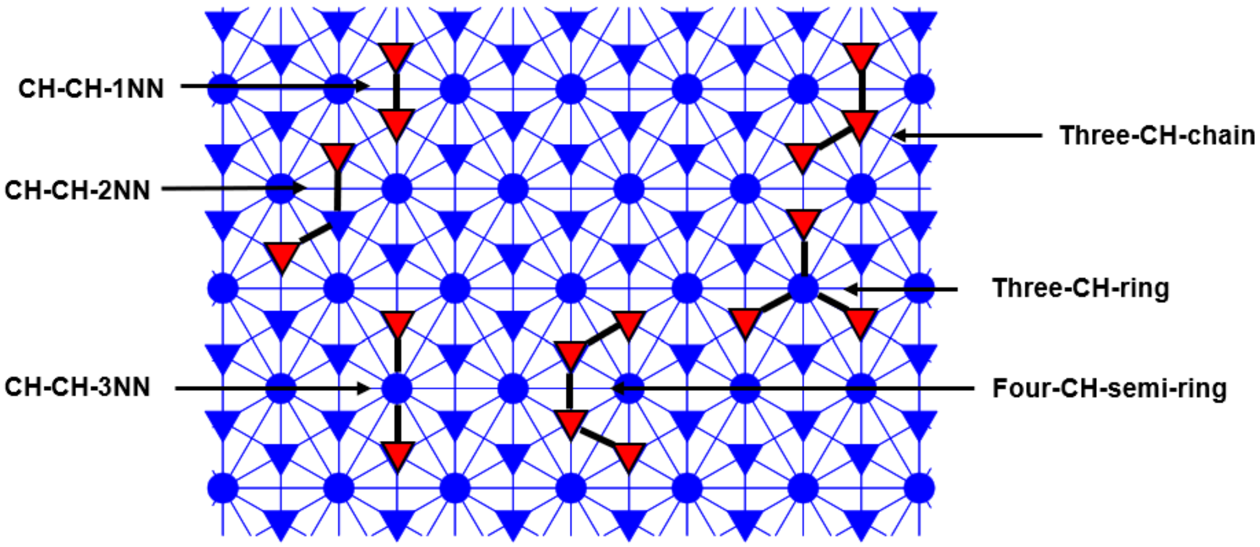

Figure S4: Schematics of interaction patterns used in the CE for capturing long-range CH configurations. The unoccupied sites are coloured in blue, whereas the occupied sites by CH are coloured in red. Each type of interaction pattern has been given an appropriate label (as shown above).

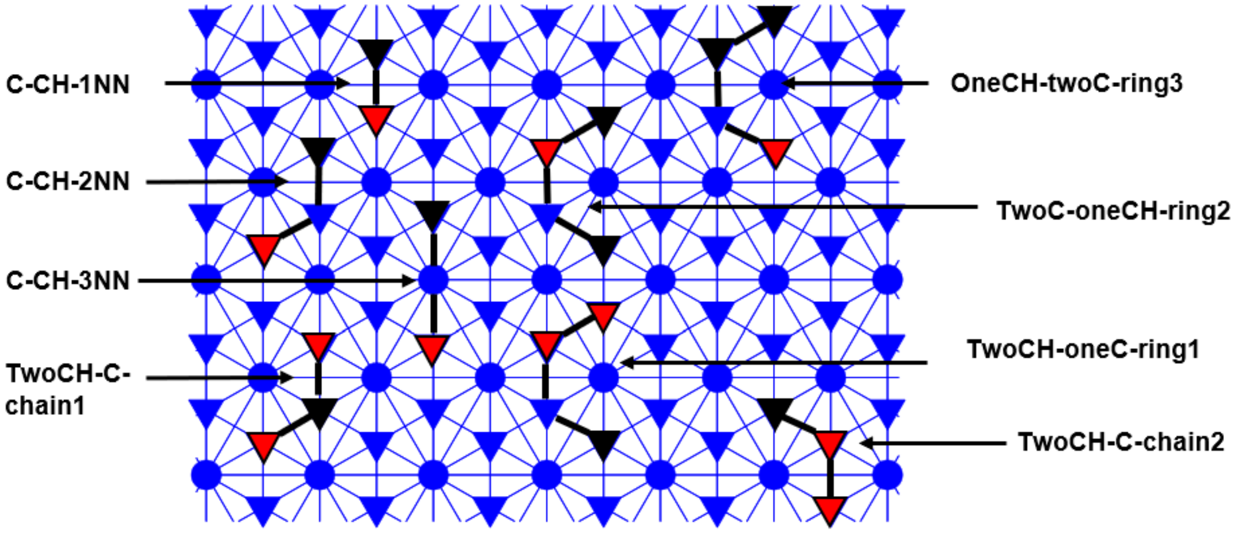

Figure S5: Schematics of interaction patterns used in the CE for capturing long-range CH configurations and CH-C configurations. The unoccupied sites are coloured in blue, whereas the occupied sites by CH and carbon are coloured in red and black, respectively. Each type of interaction pattern has been given an appropriate label (as shown above).

Table S3. Comparison of computed activation barriers of methane cracking reaction on Ni(111) with literature

Reactions	Activation energy forward (eV)	Activation energy reverse (eV)	Activation energy forward (eV) (literature values)	Activation energy reverse (eV) (literature values)
CH_4 adsorption	0.00	0.26	0.00[2]	0.02[2]
CH_4 dissociation	0.67	0.94	1.21,[3] 0.91[2]	0.66,[3] 0.90[2]
CH_3 dissociation	0.66	0.64	0.87,[3] 0.70[2]	0.54,[3] 0.63[2]
CH_2 dissociation	0.26	0.63	0.43,[3] 0.35[2]	0.59,[3] 0.69[2]
CH dissociation	1.31	0.84	1.45,[3] 1.33[2]	0.80,[3] 0.81[2]
H_2 adsorption	0.00	0.49	0.00[2]	0.22[2]
H_2 dissociation	0.04	0.89	0.06[2]	0.92[2]
CH_3 diffusion	0.21	0.21	0.15[4]	0.15[4]
CH_2 diffusion	0.26	0.26	0.19[4]	0.19[4]
CH diffusion	0.34	0.34	0.32[4]	0.32[4]
C diffusion	0.32	0.32	0.31[4]	0.31[4]
H diffusion	0.14	0.14	0.12[4]	0.12[4]

Note: The activation energies reported above do not include ZPE/thermal contributions. Ref. [2] simulation details: a p(3x3) Ni(111) supercell, ENCUT: 400.00 eV, 5×5×1 Monkhorst-Pack k-point mesh and the BEEF-vdW functional have been employed. Ref. [3] simulation details: a p(3x3) Ni(111) supercell, ENCUT: 400.00 eV, 3×3×1 Monkhorst-Pack k-point mesh and the PBE functional have been employed. Ref. [4] simulation details: a p(3x3) Ni(111) supercell, ENCUT: 415.00 eV, 7x7×1 Monkhorst-Pack k-point mesh and the BEEF-vdW functional have been employed.

The first step, methane adsorption, is not included explicitly in the KMC model because methane physisorbs weakly on the Ni(111) surface and the harmonic approximation is not appropriate for weakly bound molecules. Furthermore, experimental studies have used molecular beam techniques and high-resolution electron energy loss spectroscopy (HREELS) to show that methane binds to the Ni(111) surface by the direct dissociation mechanism. Thus, in the KMC model, we have lumped the adsorption and methane dissociation step into a single event (refer to Table 2 of the main manuscript). Similarly, we also lumped the H_2 adsorption and subsequent dissociation into a single step, since the H_2* species is short-lived on the Ni(111) surface and dissociates spontaneously into H atoms.

As shown in Table S3, using the PBE-D3 functional, we calculate the forward activation barrier for methane dissociative adsorption to be 0.67 eV. However, many theoretical studies in the literature have reported substantially high forward activation barriers for methane dissociation (refer to Table S3). Most studies have used the GGA functionals (PBE, RPBE) for studying the methane cracking reaction network. In Table S4, we report the forward activation barriers of methane dissociative chemisorption for a range of functionals and compare their values to the experimental apparent activation energies (taken from the literature). There is a significant variation in the CH_4 dissociation barrier prediction among the functionals considered. We find that the PBE-D3 functional is in reasonable agreement with the experimental apparent activation energy values of methane dissociation reported in the literature (as shown in Table S4).

Table S4. Comparison of methane dissociation activation barriers using different DFT functionals to available experimental data

Method	Methane dissociation forward activation barrier (eV)
PBE	0.84
RPBE	1.19
revPBE	1.15
PBE-D3	0.67
RPBE-D3	0.79
revPBE-D3	0.72
optB86b-vdW	0.69
optPBE-vdW	0.91
Experiment (apparent activation barrier values)	0.54[5], 0.77[6] (±0.1)

Note: The dissociation barriers reported above have been computed by considering physisorbed methane as the initial state (in the case of DFT-D3 and vdW-DF functionals).

Table S5. The geometry, event-multiplicity and proximity factors for each elementary event of methane cracking

Event	Geometry factors	Event multiplicity factors	*w*
CH_4 (g) + *(fcc) + *(top) + *(fcc) → CH_3*(fcc) + *(top) + H*(fcc)	3	2	1.00
CH_3*(fcc) + *(top) + H*(fcc) → CH_4 (g) + *(fcc) + *(top) + *(fcc)	6	1	0.00
CH_3*(fcc) + *(top) + *(fcc) → CH_2*(fcc) + *(top) + H*(fcc)	12	1	0.50
CH_2*(fcc) + *(top) + H*(fcc) → CH_3*(fcc) + *(top) + *(fcc)	12	1	0.50
CH_2*(fcc) + *(fcc) → CH*(fcc) + H*(fcc)	3	1	0.50
CH*(fcc) + H*(fcc) → CH_2*(fcc) + *(fcc)	3	1	0.50
CH*(fcc) + *(top) + *(fcc) → C*(fcc) + *(top) + H*(fcc)	6	1	0.50
C*(fcc) + *(top) + H*(fcc) → CH*(fcc) + *(top) + *(fcc)	6	1	0.50
*(fcc) + H_2 (g) + *(fcc) → H*(fcc) + *(top) + H*(fcc)	3	2	0.50
H*(fcc) + *(top) + H*(fcc) → *(fcc) + H_2 (g) + *(fcc)	3	2	0.50

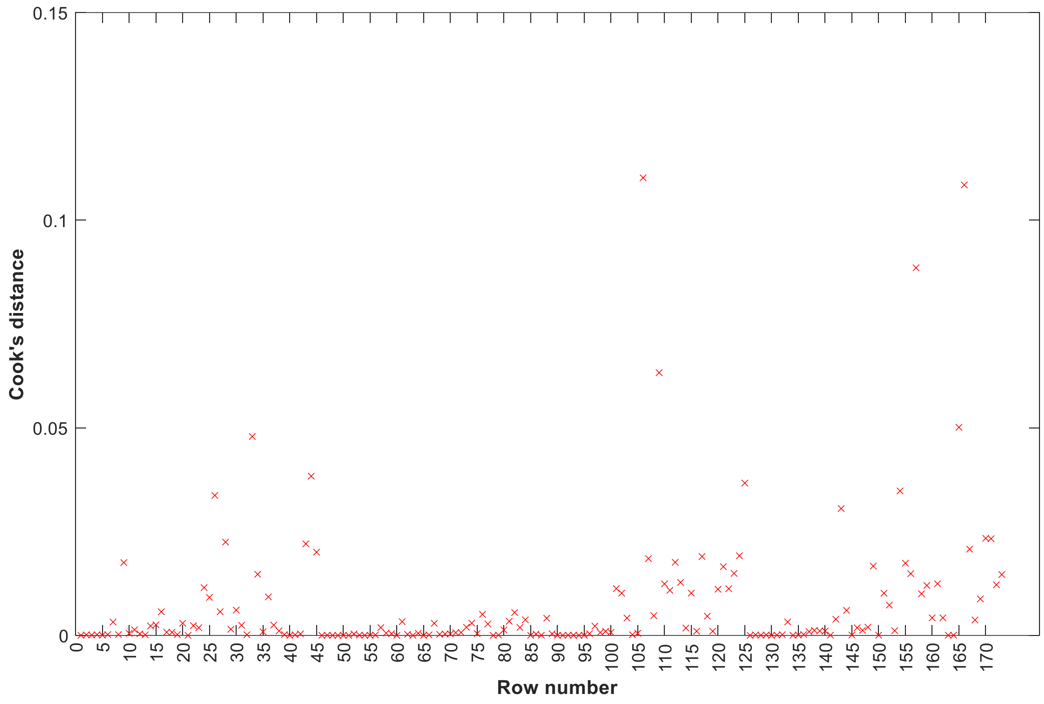

Figure S6: Cook's distances for the DFT parameterised CE model

Table S6. Effective cluster interaction (ECI) values of pairwise sub-patterns

Co-adsorbed system	ECI-1NN (eV)	ECI-2NN (eV)	ECI-3NN (eV)
$C-CH_3$	5.000	0.337	0.171
$C-CH_2$	5.000	0.270	0.099
C-CH	-0.494	0.275	0.104
C-C	-0.471	0.375	0.242
C-H	0.653	0.129	0.018
$CH-CH_3$	5.000	0.312	0.223
$CH-CH_2$	5.000	0.197	0.109
CH-CH	-0.355	0.261	0.113
CH-H	0.543	0.063	0.002
CH_2-CH_3	5.000	0.356	0.287
CH_2-CH_2	-0.366	0.161	0.125
CH_2-H	5.000	0.065	0.014
$H-CH_3$	0.534	0.089	0.055
H-H	0.268	0.004	-0.017
CH_3-CH_3	5.000	5.000	5.000

Note: The corresponding graph-pattern for every pairwise interaction parameter is depicted in Figure S2.

Table S7. Effective cluster interaction (ECI) values of the CE parameterised KMC model (referred to as "KMC-long-range")

Co-adsorbed system	ECI-1NN (eV)	ECI-2NN (eV)	ECI-3NN (eV)
C-CH_3	5.000	0.270	0.104
C-CH_2	5.000	0.203	0.032
C-CH	-0.471	0.171	0.096
C-C	-0.458	0.343	0.219
C-H	0.586	0.062	-0.050
CH-CH_3	5.000	0.276	0.187
CH-CH_2	5.000	0.162	0.007
CH-CH	-0.314	0.208	0.006
CH-H	0.508	0.027	-0.034
CH_2-CH_3	5.000	0.355	0.286
CH_2-CH_2	-0.365	0.161	0.125
CH_2-H	5.000	0.065	0.014
H-CH_3	0.534	0.089	0.055
H-H	0.268	0.004	-0.016
CH_3-CH_3	5.000	5.000	5.000
**ECI parameters of C/CH species (eV)**			
Carbon-one-body	2.320		
CH-one-body	1.162		
Carbon-three-body-chain	-0.146		
Carbon-three-body-ring	-0.313		
Carbon-four-body-square	-0.447		
Four-C-branch	-0.611		
Three-CH-chain	0.411		
Three-CH-ring	-0.065		
Four-CH-semi-ring	-0.463		
TwoCH-C-chain1	0.170		
TwoCH-C-chain2	0.227		
TwoCH-oneC-ring1	-0.414		
TwoC-oneCH-ring2	-0.196		
OneCH-twoC-ring3	-0.076		

Note: The corresponding graph pattern of CE-fit parameters are illustrated in Figures S3-S5

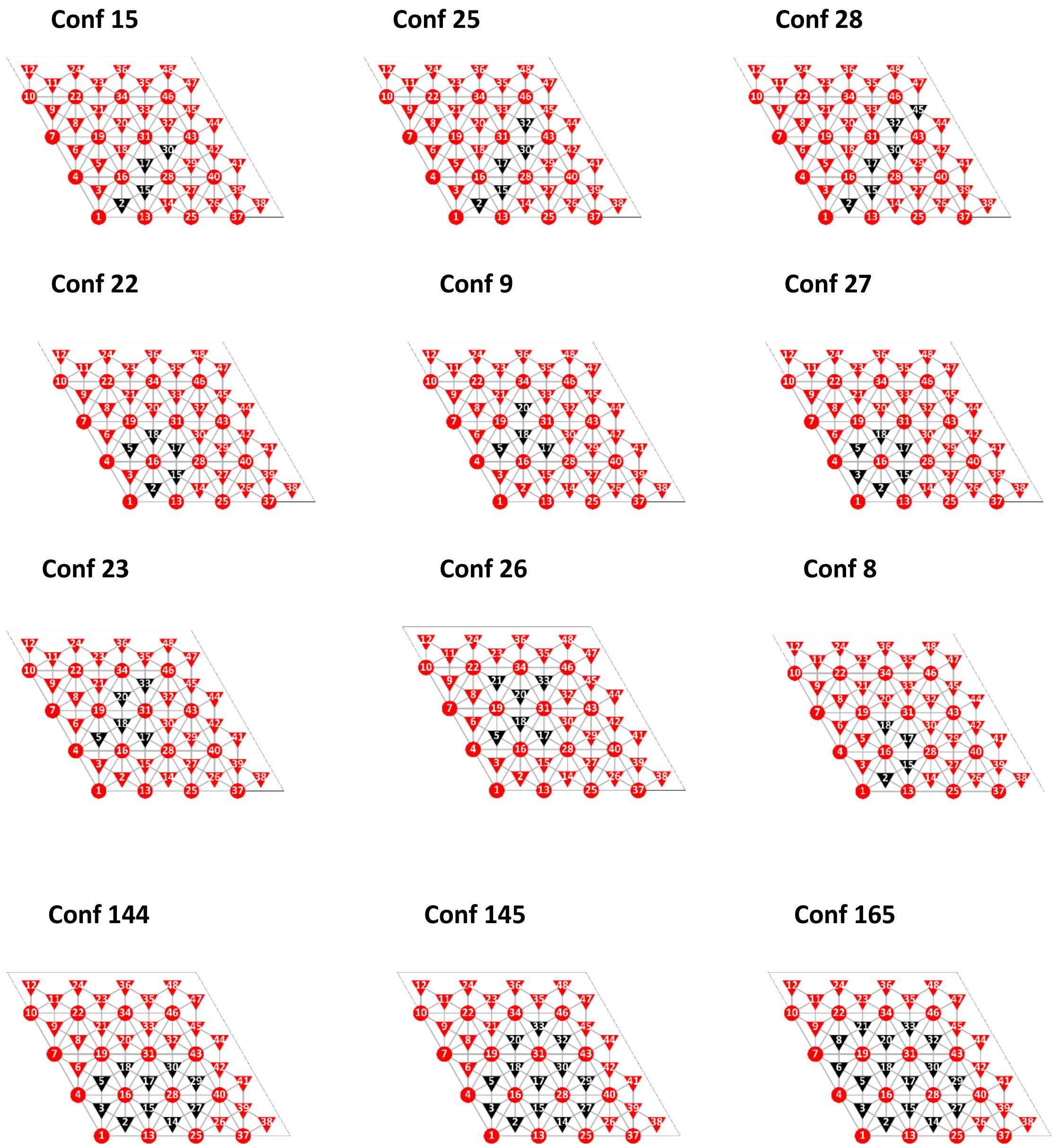

Figure S7: Schematics of a few representative/important carbon configurations of the DFT dataset (which was used for CE training with a 4×4 KMC lattice). The top and hollow sites are represented by circles and triangles, respectively. The sites coloured in red are unoccupied, whereas the sites coloured in black are occupied by carbon.

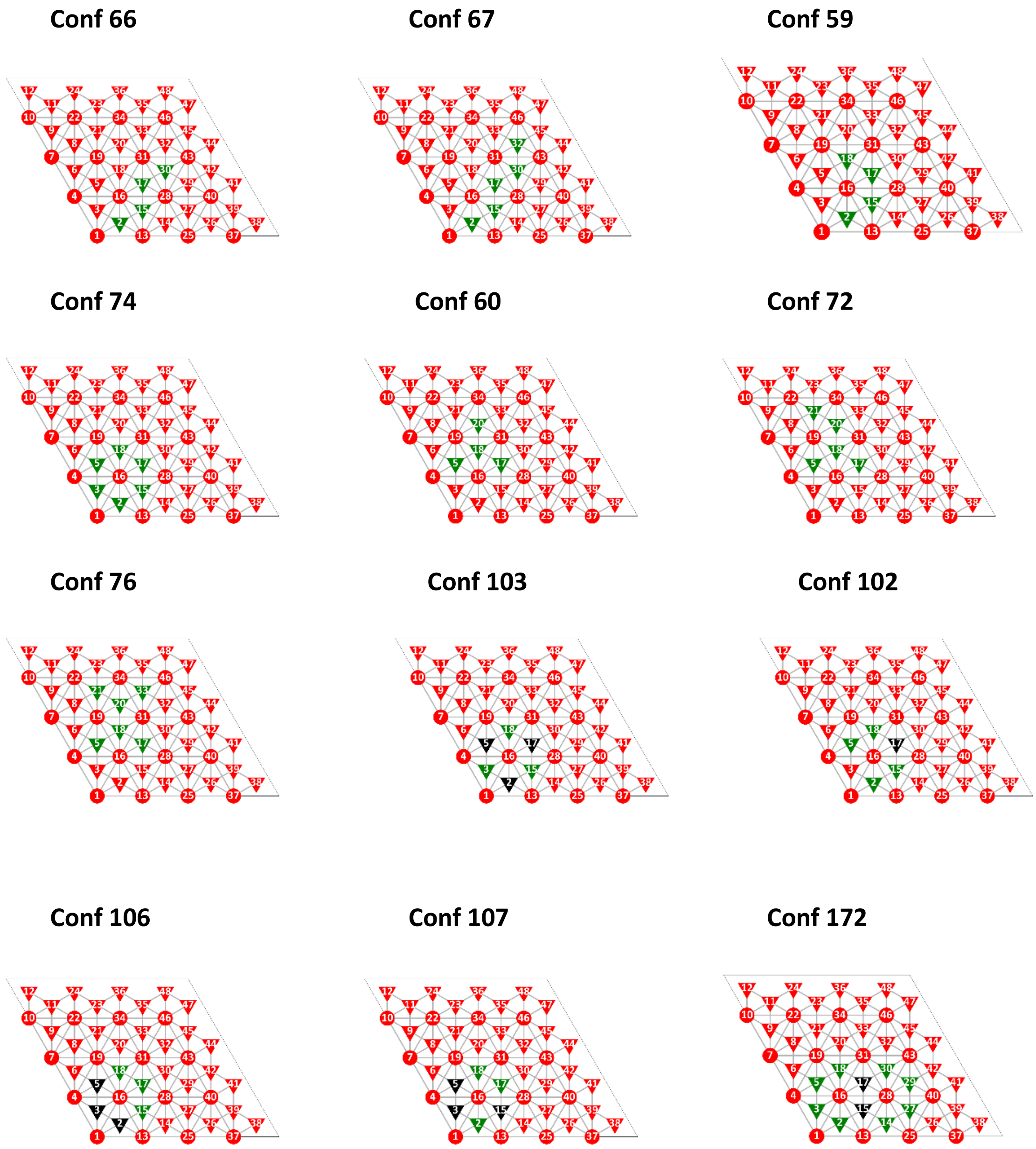

Figure S8: Schematics of a few representative/important CH and C-CH configurations of the DFT dataset (which was used for CE training with a 4×4 KMC lattice). The top and hollow sites are represented by circles and triangles, respectively. The sites coloured in red are unoccupied, whereas the sites coloured in green and black are occupied by CH and carbon, respectively.

Table S8. Results of the KMC lattice convergence test performed using KMC-1NN model at 800 K, 10.00 bar CH_4 (g) and 0.01 bar H_2 (g)

KMC lattice size	CH coverage (ML)	Carbon coverage (ML)
5x5	1.25	0.50
10x10	1.35	0.59
20x20	1.41	0.57

Table S9. Free energy and kinetic data of methane cracking reaction steps at 800 K, 10.00 bar CH_4 (g) and 0.01 H_2 (g)

Reaction	G_{fwd}^{act} (eV)	G_{rev}^{act} (eV)	G_{rxn}^{energy}(eV)	k_{fwd} (s^{-1})	k_{rev} (s^{-1})	K_{eq} (s^{-1})
CH_4 direct dissociation	1.11	0.73	0.38	1.69e+06	4.19e+08	4.04e-03
CH_3 dissociation	0.51	0.56	-0.05	1.02e+10	4.94e+09	2.07e+00
CH_2 dissociation	0.20	0.57	-0.37	9.15e+11	4.27e+09	2.14e+02
CH dissociation	1.12	0.71	0.41	1.46e+06	5.60e+08	2.61e-03
H_2 associative desorption	0.91	0.98	-0.07	3.08e+07	1.12e+07	2.76e+00

Table S10. Free energy and kinetic data of methane cracking reaction steps at 900 K, 10.00 bar CH_4 (g) and 0.01 H_2 (g)

Reaction	G_{fwd}^{act} (eV)	G_{rev}^{act} (eV)	G_{rxn}^{energy}(eV)	k_{fwd} (s^{-1})	k_{rev} (s^{-1})	K_{eq} (s^{-1})
CH_4 direct dissociation	1.20	0.71	0.49	3.57e+06	1.98e+09	1.80e-03
CH_3 dissociation	0.52	0.57	-0.05	2.29e+10	1.20e+10	1.91e+00
CH_2 dissociation	0.22	0.57	-0.35	1.10e+12	1.20e+10	9.12e+01
CH dissociation	1.12	0.70	0.42	1.00e+07	2.25e+09	4.45e-03
H_2 associative desorption	0.87	1.11	-0.24	2.52e+08	1.14e+07	2.21e+01

Table S11. Free energy and kinetic data of methane cracking reaction steps at 1000 K, 10.00 bar CH_4 (g) and 0.01 H_2 (g)

Reaction	G_{fwd}^{act} (eV)	G_{rev}^{act} (eV)	G_{rxn}^{energy}(eV)	k_{fwd} (s^{-1})	k_{rev} (s^{-1})	K_{eq} (s^{-1})
CH_4 direct dissociation	1.29	0.69	0.60	6.56e+06	6.93e+09	9.46e-04
CH_3 dissociation	0.52	0.57	-0.05	4.98e+10	2.79e+10	1.79e+00
CH_2 dissociation	0.23	0.57	-0.34	1.44e+12	2.79e+10	5.17e+01
CH dissociation	1.11	0.70	0.41	5.30e+07	6.17e+09	8.58e-03
H_2 associative desorption	0.84	1.25	-0.41	1.22e+09	1.04e+07	1.17e+02

Table S12. Free energy and kinetic data of methane cracking reaction steps at 1100 K, 10.00 bar CH_4 (g) and 0.01 H_2 (g)

Reaction	G_{fwd}^{act} (eV)	G_{rev}^{act} (eV)	G_{rxn}^{energy}(eV)	k_{fwd} (s^{-1})	k_{rev} (s^{-1})	K_{eq} (s^{-1})
CH_4 direct dissociation	1.38	0.67	0.71	1.09e+07	1.95e+10	5.58e-04
CH_3 dissociation	0.52	0.57	-0.05	9.49e+10	5.60e+10	1.69e+00
CH_2 dissociation	0.24	0.58	-0.34	1.82e+12	5.04e+10	3.61e+01
CH dissociation	1.11	0.69	0.42	1.88e+08	1.58e+10	1.19e-02
H_2 associative desorption	0.81	1.39	-0.58	4.45e+09	9.80e+06	4.54e+02

Table S13. Free energy and kinetic data of methane cracking reaction steps at 1200 K, 10.00 bar CH_4 (g) and 0.01 H_2 (g)

Reaction	G_{fwd}^{act} (eV)	G_{rev}^{act} (eV)	G_{rxn}^{energy}(eV)	k_{fwd} (s^{-1})	k_{rev} (s^{-1})	K_{eq} (s^{-1})
CH_4 direct dissociation	1.47	0.65	0.82	1.67e+07	4.65e+10	3.60e-04
CH_3 dissociation	0.52	0.57	-0.05	1.64e+11	1.01e+11	1.62e+00
CH_2 dissociation	0.25	0.58	-0.33	2.23e+12	9.15e+10	2.43e+01
CH dissociation	1.11	0.69	0.42	5.44e+08	3.16e+10	1.72e-02
H_2 associative desorption	0.78	1.53	-0.75	1.32e+10	9.37e+06	1.41e+03

References

(1) Fan, C.; Zhu, Y. A.; Yang, M. L.; Sui, Z. J.; Zhou, X. G.; Chen, D. Density Functional Theory-Assisted Microkinetic Analysis of Methane Dry Reforming on Ni Catalyst. *Ind. Eng. Chem. Res.* **2015,** *54*, 5901-5913.

(2) Zhu, Y. A.; Chen, D.; Zhou, X. G.; Yuan, W. K. DFT Studies of Dry Reforming of Methane on Ni Catalyst. *Catal. Today* **2009,** *148*, 260-267.

(3) Niu, J.; Wang, Y.; Qi, Y.; Dam, A. H.; Wang, H.; Zhu, Y.-A.; Holmen, A.; Ran, J.; Chen, D. New Mechanism Insights into Methane Steam Reforming on Pt/Ni from DFT and Experimental Kinetic Study. *Fuel* **2020,** *266*.

(4) Lozano-Reis, P.; Prats, H.; Gamallo, P.; Illas, F.; Sayós, R. Multiscale Study of the Mechanism of Catalytic CO_2 Hydrogenation: Role of the Ni(111) Facets. *ACS Catal.* **2020,** *10*, 8077-8089.

(5) Beebe, T. P.; Goodman, D. W.; Kay, B. D.; Yates, J. T. Kinetics of the Activated Dissociative Adsorption of Methane on the Low Index Planes of Nickel Single-Crystal Surfaces. *J. Chem. Phys.* **1987,** *87*, 2305-2315.

(6) Egeberg, R. C.; Ullmann, S.; Alstrup, I.; Mullins, C. B.; Chorkendoff, I. Dissociation of CH_4 on Ni(111) and Ru(0001). *Surf. Sci.* **2002,** *497*, 183-193.
